# The Current State and Validity of Digital Assessment Tools for Psychiatry: Systematic Review

**DOI:** 10.2196/32824

**Published:** 2022-03-30

**Authors:** Nayra A Martin-Key, Benedetta Spadaro, Erin Funnell, Eleanor Jane Barker, Thea Sofie Schei, Jakub Tomasik, Sabine Bahn

**Affiliations:** 1 Cambridge Centre for Neuropsychiatric Research Department of Chemical Engineering and Biotechnology University of Cambridge Cambridge United Kingdom; 2 University of Cambridge Medical Library University of Cambridge Cambridge United Kingdom; 3 Psyomics Ltd Cambridge United Kingdom

**Keywords:** diagnostic accuracy, digital mental health, digital questionnaire, psychiatry, systematic review

## Abstract

**Background:**

Given the role digital technologies are likely to play in the future of mental health care, there is a need for a comprehensive appraisal of the current state and validity (ie, screening or diagnostic accuracy) of digital mental health assessments.

**Objective:**

The aim of this review is to explore the current state and validity of question-and-answer–based digital tools for diagnosing and screening psychiatric conditions in adults.

**Methods:**

This systematic review was based on the Population, Intervention, Comparison, and Outcome framework and was carried out in accordance with the PRISMA (Preferred Reporting Items for Systematic Reviews and Meta-Analyses) guidelines. MEDLINE, Embase, Cochrane Library, ASSIA, Web of Science Core Collection, CINAHL, and PsycINFO were systematically searched for articles published between 2005 and 2021. A descriptive evaluation of the study characteristics and digital solutions and a quantitative appraisal of the screening or diagnostic accuracy of the included tools were conducted. Risk of bias and applicability were assessed using the revised tool for the Quality Assessment of Diagnostic Accuracy Studies 2.

**Results:**

A total of 28 studies met the inclusion criteria, with the most frequently evaluated conditions encompassing generalized anxiety disorder, major depressive disorder, and any depressive disorder. Most of the studies used digitized versions of existing pen-and-paper questionnaires, with findings revealing poor to excellent screening or diagnostic accuracy (sensitivity=0.32-1.00, specificity=0.37-1.00, area under the receiver operating characteristic curve=0.57-0.98) and a high risk of bias for most of the included studies.

**Conclusions:**

The field of digital mental health tools is in its early stages, and high-quality evidence is lacking.

**International Registered Report Identifier (IRRID):**

RR2-10.2196/25382

## Introduction

### Background

Mental health disorders are highly prevalent [1] and represent the main source of health-related economic burden worldwide [2-4], with barriers to ensuring adequate mental health care provision being complex and multifaceted. For instance, in addition to the lack of available mental health care professionals worldwide [5], short primary care consultation times coupled with the complexity and subjectivity of diagnosing mental health disorders mean that many patients are not receiving adequate support. Furthermore, attitudinal factors, including a low perceived treatment need and a fear of stigmatization, contribute significantly to non–help-seeking behavior [6]. Moving forward, there is a need for innovative, cost-effective, and highly scalable solutions for the assessment, diagnosis, and management of mental health disorders.

To this end, digital technologies for psychiatry may offer attractive *add-ons* or alternatives to conventional mental health care services. Clinical decision support tools may range from simple digitized versions of existing pen-and-paper mental health screening instruments to more sophisticated question-and-answer–based digital solutions for psychiatry such as adaptive questionnaires. Given the ubiquitous nature of technology, these tools can be used on patients’ personal devices, such as via a website, thereby offering private and convenient mental health care provision from the comfort of one’s home.

Critically, although there exists a plethora of research evaluating digital psychotherapeutic technologies such as internet-delivered cognitive behavioral therapy [7,8], to our knowledge, little effort has been put into evaluating diagnostic decision support technologies. The limited number of studies on diagnostic and screening tools for mental health have mainly focused on establishing the psychometric properties of digitized versions of existing pen-and-paper questionnaires (see van Ballegooijen et al [9] for a systematic review) and have often compared these tools to existing scales such as the 9-item Patient Health Questionnaire (PHQ–9) [10] as opposed to a *gold standard* assessment by a psychiatrist or a diagnostic interview based on the Diagnostic and Statistical Manual of Mental Disorders (DSM; now in its fifth edition [DSM–5]) [11] or the International Statistical Classification of Diseases and Related Health Problems (ICD; now in its 11th edition [ICD–11]) [12,13]. In fact, despite the rapidly growing number of digital assessment tools for screening and diagnosing mental health disorders, little is known about their accuracy.

### Objectives

To this end, the key objectives of this systematic review are to summarize available digital mental health assessment tools as well as evaluate their accuracy among studies using a *gold standard* reference test. We will first examine the types of available digital mental health assessment tools (eg, digitized versions of existing psychiatric pen-and-paper questionnaires vs more sophisticated digital tools). Second, we will evaluate the screening or diagnostic accuracy of the identified digital mental health assessment tools for each mental health condition of interest. Finally, we will assess the risk of bias and applicability of all the included studies. Given the rapid pace of technological development and the role digital technologies are likely to play in the future of mental health care, this comprehensive systematic review is timely and has important implications for clinical practice and the development of digital solutions for psychiatry.

## Methods

### Database Search

The methods are described in detail in a previously published protocol [14], which has been registered with the International Prospective Register of Systematic Reviews (PROSPERO CRD42020214724). The search strategy was developed using the Population, Intervention, Comparison, and Outcome framework and performed following the PRISMA (Preferred Reporting Items for Systematic Reviews and Meta-Analyses [15]) guidelines. Keywords and subject headings were extracted from a preliminary scan of the literature and the DSM–5 and ICD–11 (or DSM–IV and ICD–10 for older publications) diagnostic manuals and were decided in consultation with a medical librarian (EJB) and a practicing psychiatrist (SB). The following electronic databases were searched: MEDLINE, Embase, Cochrane Library, ASSIA, Web of Science Core Collection, CINAHL, and PsycINFO. Search terms were grouped into four themes and combined using the following structure: “digital technology” *AND* “assessment tool” *AND* “mental health” *AND* “accuracy.” The search was completed on October 12, 2021. Gray literature (eg, clinical trial databases, unpublished theses, reports, and conference presentations) was identified by hand searching. Other potentially eligible publications were identified by hand searching the reference lists of relevant systematic reviews and meta-analyses. Hand searching was completed on October 21, 2021. A complete list of the search strategies, including keywords and subject headings, can be found in Multimedia Appendix 1.

### Inclusion and Exclusion Criteria

Owing to ongoing developments in the digitization of existing psychiatric questionnaires and the rapid growth in digital assessment tools for the screening and diagnosing of mental health conditions, the initial search was limited to studies published between January 1, 2005, and October 12, 2021, with hand searching completed by October 21, 2021. Studies published in any language were included. The study design was not limited to ensure that no relevant studies were missed.

The population included adults with a mean age of 18 to 65 years who had been assessed for the presence of any of the following mental health conditions: bipolar disorder (BD), major depressive disorder (MDD), anxiety disorders, obsessive-compulsive disorder (OCD), insomnia, schizophrenia, attention-deficit/hyperactivity disorder (ADHD), autism spectrum disorders, eating disorders, personality disorders, alcohol use disorder (AUD), substance use disorder (SUD), posttraumatic stress disorder (PTSD), acute stress disorder, and adjustment disorder. In addition to these conditions, notable symptom domains such as self-harm, suicidality, and psychosis were included based on their relevance in psychiatric assessments. The population included any gender, severity of mental health concern, ethnicity, and geographical location.

As the review focused on the screening or diagnostic accuracy of digital mental health assessments for use in the primary care or general and psychiatric populations, specific subgroups such as pregnant individuals, refugee or asylum seekers, prisoners, and those in acute crisis or admitted to emergency services were excluded. In consultation with a practicing psychiatrist (SB), we also excluded studies on somatoform disorders and specific phobias as these are less frequently diagnosed in primary care and rarely present in secondary care. Studies on tools used to identify neuropsychiatric disorders (eg, dementias) or any disorders that are due to clinically confirmed temporary or permanent dysfunction of the brain were outside the scope of the review. In addition, studies on tools used to identify mental health disorders in physical illnesses (eg, cancer) were excluded.

The interventions targeted in this review included question-and-answer–based digital mental health screening or diagnostic tools completed by the patient. Studies of digital assessment tools that were not *exclusively* question-and-answer–based, such as blood tests, imaging techniques, monitoring tools, genome analyses, accelerometer devices, and wearables, were excluded. Furthermore, studies on digital assessment tools used to predict future risk of developing a mental health disorder were also excluded, except in the case of suicidality.

Only studies that evaluated the accuracy of a digital mental health assessment tool against a *gold standard* reference test, such as an assessment by a psychiatrist or a standardized structured or semistructured interview based on the DSM–5 and ICD–11 criteria (or DSM–IV and ICD–10 for older publications), were included. Studies that did not include an outcome measure of accuracy (eg, sensitivity and specificity or area under the receiver operating characteristic curve [AUC]) were not included.

### Outcomes Measured

The primary outcome was to examine the current state of digital mental health assessment tools, including the type of tools being used (eg, digitized versions of existing psychiatric pen-and-paper questionnaires) and targeted conditions. The secondary outcome was the validity (ie, screening or diagnostic accuracy) of the identified digital mental health assessment tools.

### Screening and Study Selection

**Figure 1:**
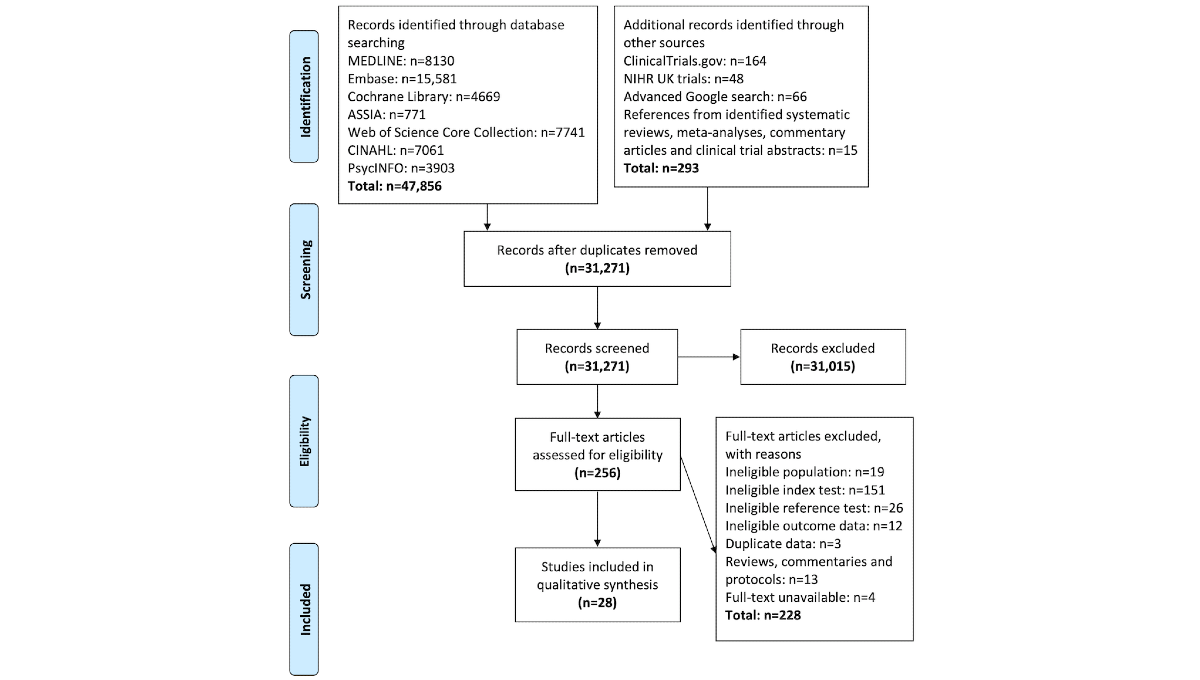
PRISMA (Preferred Reporting Items for Systematic Reviews and Meta-Analyses) flowchart of included studies. NIHR: National Institute for Health Research.

Articles identified from the database searches were first stored in the reference management software package EndNote (Clarivate Analytics), which was used to eliminate any duplicates. Once duplicates had been eliminated, all identified articles were transferred to the systematic review software Rayyan (Rayyan Systems Inc). In total, 2 independent reviewers (BS and EF) screened the titles and abstracts of all the studies. Any disagreements were discussed and resolved with a third reviewer (NAM-K). Full texts were then retrieved for the included studies and subsequently assessed for relevance against the eligibility criteria by the 2 independent reviewers. In addition, the full texts of any studies that did not specify in the title or abstract whether the tools used were digital or pen-and-paper versions were examined by the 2 independent reviewers. Once again, any disagreements were discussed and resolved with the third reviewer. Reasons for inclusion and exclusion were recorded at the full-text screening stage and are shown in Figure 1.

### Study Characteristics

A descriptive evaluation of the study characteristics, including conditions of interest, sample type and size, proportion of women, mean age, and country, was extracted by the 2 independent reviewers and summarized.

### Digital Mental Health Assessments and Their Validity Per Condition

Information regarding the digital mental health assessments (ie, index tests), including the type and number of questions, reference tests, time flow, and blinding, was extracted by the 2 independent reviewers and summarized. In addition, a descriptive appraisal of the screening or diagnostic accuracy of the included digital mental health assessment tools separated by condition of interest was conducted. The following values were extracted or calculated based on the available data for each digital tool separated by condition of interest:

- Sensitivity: the capacity of the digital tool to correctly classify those with the condition
- Specificity: the capacity of the digital tool to correctly classify those without the condition
- Youden index: a single statistic that measures the performance of a dichotomous diagnostic test at a given cutoff and can be used for maximizing sensitivity and specificity, with scores ranging from 0 (not useful) to 1 (perfect)
- AUC: shows the degree of separability between 2 conditions and represents the probability that a randomly selected individual with the condition is rated or ranked as more likely to have the condition than a randomly selected individual without the condition (≥0.9=excellent, ≥0.8=good, ≥0.7=fair, ≥0.6=poor, ≥0.5=fail [16])

Given the wide range of digital mental health assessment tools and cutoffs used and the differences in methodology and patient populations, as well as the lack of available raw data (after having contacted the authors for further details), a meta-analysis was not deemed clinically informative at this stage.

### Risk of Bias and Applicability Assessment

The 2 independent reviewers assessed the risk of bias and applicability of all the included studies using the revised tool for the Quality Assessment of Diagnostic Accuracy Studies 2 (QUADAS–2 [17]), which is recommended for use in systematic reviews of diagnostic accuracy by the United Kingdom National Institute for Health and Clinical Excellence and the Agency for Healthcare Research and Quality, Cochrane Collaboration [18]. Any disagreements were discussed and resolved with the third reviewer. The developers of the QUADAS–2 tool recommend that the tool be tailored for each speciﬁc review by adding or omitting signaling questions, which are included to assist in judgments about risk of bias. To this end, the following question was omitted: *Did all patients receive a reference standard?* The reason for removing this question was based on the fact that screening and diagnostic test accuracy studies in the field of mental health ordinarily provide the reference standard to a subset of the original sample, primarily because of missing data by study design or clinical practice [19]. It was agreed that this question was overly conservative for this review. In light of this amendment, we rephrased the following question—*Were all patients included in the analysis?—*to *Did the data analysis only include patients who received both the index test and the reference standard?*

## Results

### Included Studies

In total, 31,271 articles were retrieved, of which 256 (0.82%) were selected for full-text review. Of these 256 articles, 28 (10.9%) were identified for inclusion. The reasons for exclusion at the full-text review stage are outlined in Figure 1.

### Study Characteristics

The characteristics of the 28 included studies are summarized in Table 1 (refer to Multimedia Appendix 2 [20-47] for a checklist summary of the mental health disorders investigated in the included studies). Notably, a large proportion of studies did not meet the inclusion criteria. This was primarily due to the studies not using a *digital* index test or appropriate reference test (ie, an assessment by a psychiatrist or a diagnostic interview based on the DSM or ICD criteria). Other exclusions regarded studies focusing on ineligible populations (eg, children or specific subgroups such as pregnant individuals, refugee or asylum seekers, prisoners, and those in acute crisis or admitted to emergency services) as well as studies that did not include an outcome measure of accuracy (eg, sensitivity and specificity or AUC).

Most of the studies included participants from primary care services or the general population (18/28, 64% [20,22-25,28,32,35,37-45,47]). This was followed by the inclusion of participants from secondary care or specialist services, including psychiatric outpatients (12/28, 43% [20,27,29-31,33-35,38,45-47]). Of the 28 studies, 6 (21%) included university students [21,23-26,36], whereas 4 (14%) purposely recruited nonpsychiatric controls [29-31,33].

Sample sizes ranged from 100 [44] to 6361 [45], with all but 3 studies [26,27,33] including a larger proportion of women. The mean age across studies ranged from 20 [26] to 53 years [44], although not all studies provided this information. Most of the included studies were conducted in the United States (12/28, 43% [20,27-34,37,43,44]). Of the 28 studies, 6 (21%) were conducted in the Netherlands [23-25,38,45,46], and 4 (14%) took place in Spain [21,22,39,42]. The remaining 6 studies (6/28, 21%) were conducted in Australia (1/28, 4%) [40], China (1/28, 4%) [26], Denmark (1/28, 4%) [41], South Korea (2/28, 7%) [35,47], and Thailand (1/28, 4%) [36].

**Table 1:** Characteristics of the included studies, including conditions of interest, sample type and size, proportion of women, mean age, and country.

Study	Conditions	Occurrence of conditions	Sample					Sample size, N		Women, n		Age (years)		Country
			Primary care or general population	Secondary care	Nonpsychiatric controls	University students								
Achtyes et al [20]^a^	MDD^b^	Current and lifetime	✓	✓			145		79		—^c^		United States	
Ballester et al [21]	Any mood disorder^d^, any anxiety disorder^e^, any depressive disorder^f^, panic disorder, GAD^g^	Current and lifetime				✓	Total: 575; interviewed: 287		Total: 55		—		Spain	
Cano-Vindel et al [22]	MDD, GAD	Current	✓				Total: 1052; interviewed: 178		Total: 77; interviewed: 70		—		Spain	
Donker et al [23]	Any depressive disorder^h^, GAD, social phobia, panic disorder, agoraphobia, OCD^i^, PTSD^j^, AUD^k^	Current	✓			✓	Total: 502; interviewed: 157		Total: 57		Total: mean 43 (SD 13)		Netherlands	
Donker et al [24]	Any depressive disorder^h^	Current	✓			✓	Total: 502; interviewed: 157		Total: 57		Total: mean 43 (SD 13)		Netherlands	
Donker et al [25]	Any depressive disorder^h^, any anxiety disorder^l^, GAD, panic disorder, social phobia, PTSD	Current	✓			✓	Total: 502; interviewed: 157		Total: 57		Total: mean 43 (SD 13)		Netherlands	
Du et al [26]	MDD	Current				✓	Total: 230; interviewed: 150		Total: 44		Total: mean 20 (SD 3)		China	
Fowler et al [27]	EUPD^m^	Current		✓			Sample 1: 653; sample 2: 1000		Sample 1: 51; sample 2: 46		Sample 1: mean 36 (SD 15); sample 2: mean 34 (SD 15)		United States	
Gaynes et al [28]	Any mood or anxiety disorder^n^, any anxiety disorder^o^, any depressive disorder^p^, bipolar spectrum disorder, PTSD	Current; lifetime only for bipolar spectrum disorder	✓				723		60		Mean 46		United States	
Gibbons et al [29]	Any depressive disorder^q^, MDD	Current		✓	✓		Total: 1605; interviewed: 292		Total: 70		Total: median 40-49		United States	
Gibbons et al [30]	MDD	Current		✓	✓		Total: 657; interviewed: 259		Total: 65		—		United States	
Gibbons et al [31]	MDD, GAD	Current		✓	✓		Total: 1614; interviewed: 387		Total: 70		Total: median 40-49		United States	
Graham et al [32]	MDD, GAD	Current	✓				269		71		Mean 57		United States	
Guinart et al [33]	Psychosis	Current		✓	✓		Total: 200; interviewed: 79		Total: 44		Total: median 30		United States	
Kertz et al [34]	GAD	Current		✓			Total: 232; interviewed: 218		Total: 60		Total: mean 35 (SD 13)		United States	
Kim et al [35]	GAD	Current	✓	✓			527		65		Mean 39 (SD 15)		South Korea	
Lohanan et al [36]	EUPD	Current				✓	Total: 342; interviewed: 68		Total: 81		Total: mean 20 (SD 1)		Thailand	
McNeely et al [37]	AUD, SUD^r^	Current	✓				Total: 462; interviewed: 459		Total: 52		Total: mean 46 (SD 12)		United States	
Meuldijk et al [38]	Any depressive disorder^s^, GAD, panic disorder, social phobia, OCD, PTSD, agoraphobia, AUD	Current	✓	✓			1292		61		Mean 40 (SD 13)		Netherlands	
Munoz-Navarro et al [39]	GAD	Current	✓				Total: 260; interviewed: 178		Total: 72; interviewed: 70		—		Spain	
Nguyen et al [40]	MDD, GAD, social phobia, panic disorder, PTSD, OCD, BN^t^, AUD	Current	✓				Total: 616; interviewed: 158		Total: 72; interviewed: 73		Total: mean 40 (SD 12)		Australia	
Nielsen et al [41]	MDD	Current	✓				Total: 246; interviewed: 152		Total: 60; interviewed: 59		Total: mean 37 (SD 13); interviewed: mean 34 (SD 13)		Denmark	
Oromendia et al [42]	Panic disorder	Current	✓				171		61		Mean 36 (SD 9)		Spain	
Rogers et al [43]	Any depressive disorder^p^, GAD, social phobia, panic disorder, BD^u^, ADHD^v^, SUD, suicidality	Current	✓				234		64		Mean 47 (SD 16)		United States	
Sanchez et al [44]	AUD	Current	✓				100		66		Mean 53 (SD 12)		United States	
Schulte-van Maaren et al [45]	Any anxiety disorder^w^	Current	✓	✓			Psychiatric outpatients: 5066; general population: 1295		Psychiatric outpatients: 64; general population: 63		Psychiatric outpatients: mean 37 (SD 12); general population: mean 40 (SD 13)		Netherlands	
Ter Huurne et al [46]	AN^x^, BN, BED^y^, EDNOS^z^	Current		✓			134		88		Mean 31 (SD 11)		Netherlands	
Yoon et al [47]	Suicidality	Current	✓	✓			528		65		No risk group: mean 39 (SD 15); risk-positive group: mean 38 (SD 15)		South Korea	

^a^The authors also looked at generalized anxiety disorder and bipolar disorder, but no accuracy data were reported.

^b^MDD: major depressive disorder.

^c^Missing data.

^d^Major depressive episode or mania or hypomania.

^e^Panic disorder or generalized anxiety disorder.

^f^Major depressive episode (unspecified).

^g^GAD: generalized anxiety disorder.

^h^MDD, dysthymia, or minor depression.

^i^OCD: obsessive-compulsive disorder.

^j^PTSD: posttraumatic stress disorder.

^k^AUD: alcohol use disorder.

^l^GAD, panic disorder, social phobia, or PTSD.

^m^EUPD: emotionally unstable personality disorder (also known as borderline personality disorder).

^n^MDD, bipolar depression, bipolar spectrum disorder, GAD, agoraphobia, panic disorder, social phobia, PTSD, or OCD.

^o^GAD, agoraphobia, panic disorder, social phobia, PTSD, or OCD.

^p^MDD or bipolar depression.

^q^MDD or minor depression.

^r^SUD: substance use disorder.

^s^Depression (unspecified) or dysthymia.

^t^BN: bulimia nervosa.

^u^BD: bipolar disorder.

^v^ADHD: attention-deficit/hyperactivity disorder.

^w^Anxiety disorder (unspecified).

^x^AN: anorexia nervosa.

^y^BED: binge eating disorder.

^z^EDNOS: eating disorder not otherwise specified.

### Digital Mental Health Assessments and Their Validity Per Condition

#### Overview

The characteristics of the 28 included studies are summarized in Table 2. None of the included studies targeted schizophrenia, autism spectrum disorders, acute stress disorder, adjustment disorder, or self-harm. Insomnia was considered by Nguyen et al [40], but the reference standard used did not meet our eligibility criteria as it did not comprise an assessment by a psychiatrist or a diagnostic interview based on the DSM or ICD criteria. Regarding screening or diagnostic accuracy, below we summarize sensitivity, specificity, and AUCs per tool by condition of interest, where available. For simplicity, where multiple cutoffs were provided for a particular tool, only sensitivity and specificity scores that resulted in the highest Youden index were presented. In the event of multiple sensitivity and specificity values being associated with an equivalent (and highest) Youden index, the values resulting in the smallest difference (ie, sensitivity-specificity) were reported (see Multimedia Appendix 3 [20-47] for sensitivity and specificity values per cutoff score as well as Youden index values and AUCs).

**Table 2:** Characteristics of the included studies, including conditions of interest, index tests, type and number of questions, reference tests, time flow, and blinding.

Study	Conditions	Index tests	Type of questions	Questions, N	Reference tests	Time flow	Blinded to index test
Achtyes et al [20]^a^	MDD^b^	CAD–MDD^c,d^	Based on existing questionnaires, DSM–IV^e^, and an expert panel	389	SCID–I^f^, DSM–IV–TR^g^	?^h^	?
Ballester et al [21]	Any mood disorder^i^, any anxiety disorder^j^, any depressive disorder^k^, panic disorder, GAD^l^	WMH–ICS^m^ surveys	Based on existing questionnaires	291	Spanish MINI^n^ (version 5.0 and 6.0), DSM–IV–TR	Within 4 weeks	✓^o^
Cano-Vindel et al [22]	MDD, GAD	PHQ–2^p^, GAD–2^q^	Digital versions of existing questionnaires	PHQ–2=2; GAD–2=2	CIDI^r^ GAD module, SCID–I, DSM–IV	?	?
Donker et al [23]	Any depressive disorder^s^, GAD, social phobia, panic disorder, agoraphobia, OCD^t^, PTSD^u^, AUD^v^	WSQ^w^, GAD–7^x^, CES–D^y^, PDSS^z^, FQ^aa^, IES–R^ab^, YBOCS^ac^, AUDIT^ad^	Based on existing questionnaires, MINI, and AUDIT; digital versions of existing questionnaires	WSQ=15; GAD–7=7; CES–D=20; PDSS=7; FQ=15; IES–R=15; YBOCS=10; AUDIT=10	Lifetime version 2.1 of the CIDI Dutch version, DSM–IV	Mean of 13 days	✓
Donker et al [24]	Any depressive disorder^s^	SID^ae^, CES–D, and K10^af^	Digital versions of existing questionnaires	SID=1; CES–D=20; K10=10	Lifetime version 2.1 of the CIDI Dutch version, DSM–IV	Mean of 13 days	?
Donker et al [25]	Any depressive disorder^s^, any anxiety disorder^ag^, GAD, panic disorder, social phobia, PTSD	GAD–7, GAD–2, GAD–SI^ah^, CES–D	Digital versions of existing questionnaires	GAD–7=7; GAD–2=2; GAD–SI=1; CES–D=20	Lifetime version 2.1 of the CIDI Dutch version, DSM–IV	Mean of 13 days	✓
Du et al [26]	MDD	PHQ–9^ai^	Digital version of existing questionnaire	9	MINI (version 5.0, Chinese depression modules), DSM–IV	Within 48 hours	✓
Fowler et al [27]	EUPD^aj,ak^	PID–5^al^, FFM^am^, SCID–II–PQ^an^	Digital versions of existing questionnaires	PID–5=220; FFM=44; SCID–II–PQ=15	SCID–II^ao^, DSM–IV	Within 72 hours	?
Gaynes et al [28]	Any mood or anxiety disorder^ap^, any anxiety disorder^aq^, any depressive disorder^ar^, bipolar spectrum disorder, PTSD	M-3^as^	Questions generated by a panel of mental health clinicians and researchers	27	MINI (version 5.0), DSM–IV	Same day or within 30 days	✓
Gibbons et al [29]	Any depressive disorder^at^, MDD	CAT–DI^c,au^	Based on existing questionnaires, DSM–IV, and an expert panel	389	SCID–I, DSM–IV, DSM–IV appendix B (for minor depression)	?	×^av^
Gibbons et al [30]	MDD	CAD–MDD^c^	Based on existing questionnaires, DSM–IV, and an expert panel	88	SCID–I, DSM–IV–TR	?	?
Gibbons et al [31]	MDD, GAD	CAT–ANX^c,aw^, CAT–DI^c^	Based on existing questionnaires, DSM–IV, and an expert panel	CAT–ANX=431; CAT–DI=389	SCID–I, DSM–IV	?	?
Graham et al [32]^ax^	MDD, GAD	CAD–MDD^c^, CAT–ANX^c^	Based on existing questionnaires, DSM–IV, and an expert panel	CAD–MDD=389; CAT–ANX=431	SCID–I, DSM–5	Same day	✓
Guinart et al [33]	Psychosis	CAT–Psychosis^c,ay^	Based on existing questionnaires and clinician-rated measures	144	SCID–I, DSM–5	Same day if not completed within last 12 months	?
Kertz et al [34]	GAD	GAD–7	Digital version of existing questionnaire	7	MINI (version 6.0), DSM–IV	?	?
Kim et al [35]	GAD	MHS: A^az^	Based on existing questionnaires and diagnostic criteria, focus group interviews with patients with GAD, and an expert panel	11	MINI (version 5.0), DSM–IV	?	✓
Lohanan et al [36]	EUPD	SI–Bord^ba^	Based on SCID–II criteria	5	SCID–II, DSM–IV	?	?
McNeely et al [37]	AUD, SUD^bb^	SISQs^bc^ for alcohol and drugs	Digital version of existing interviewer-administered SISQs	SISQ−alcohol=1; SISQ−drugs=1	MINI–Plus (version 6.0), DSM–IV	Same day	?
Meuldijk et al [38]	Any depressive disorder^bd^, GAD, panic disorder, social phobia, OCD, PTSD, agoraphobia, AUD	WSQ	Based on existing questionnaire, MINI, and AUDIT	15	MINI–Plus (version 5.0), DSM–IV–TR	?	?
Munoz-Navarro et al [39]	GAD	GAD–7	Digital version of existing questionnaire	7	CIDI GAD module Spanish version, DSM–IV	?	✓
Nguyen et al [40]	MDD, GAD, social phobia, panic disorder, PTSD, OCD, BN^be^, AUD	e-PASS^c,bf^	Based on the DSM–IV–TR criteria; includes a variety of demographic questions	>540	MINI–Plus (version 5.0), DSM–IV, ADIS–IV^bg^ (if anxiety symptoms present), DSM–IV–TR	Mean of 10.5 (range 1-34) days	✓
Nielsen et al [41]	MDD	MDI^bh^	Digital version of existing questionnaire	13	M–CIDI^bi^ computerized Norwegian version, DSM–IV	Within 2 weeks	✓
Oromendia et al [42]	Panic disorder	WSQ	Based on existing questionnaire	1	SCID–I, DSM–IV	Mean of 14 days	?
Rogers et al [43]	Any depressive disorder^ar^, GAD, social phobia, panic disorder, BD^bj^, ADHD^bk^, SUD, suicidality	CMFC^bl^ (initial screener and SAMs^bm^)	Expert panel	Initial screener=8; SAMs=11-27	SCID–5–RV^bn^, DSM–5	Same day	✓
Sanchez et al [44]	AUD	TAPS–1^bo^	Based on the NIDA^bp^ Quick Screen version 1.0	4	CIDI Spanish version, DSM–5	Same day	?
Schulte-van Maaren et al [45]	Any anxiety disorder^bq^	BSA^br^, PI–R^bs^, PAI^bt^, PSWQ^bu^, WDQ^bv^, SIAS^bw^, SPS^bx^, IES–R	Digital versions of existing questionnaires	BSA=10; PI–R=41; PAI=15; PSWQ=16; WDQ=30; SIAS=20; SPS=20; IES–R=22	MINI–Plus (version 5.0), DSM–IV	?	?
Ter Huurne et al [46]	AN^by^, BN, BED^bz^, EDNOS^ca^	EDQ–O^cb^	Based on MINI–Plus and DSM–IV–TR criteria	26	Clinical interview based on the DSM–IV–TR criteria	Mean of 9 days (range of several hours to 48 days)	✓
Yoon et al [47]	Suicidality	UBCS^cc^	Literature review and expert panel	12	MINI (version 5.0), DSM–IV	Same day	✓

^a^The authors also used the Computerized Adaptive Test–Depression Inventory, Computerized Adaptive Test–Anxiety, and Computerized Adaptive Test–Mania, but no accuracy data were reported.

^b^MDD: major depressive disorder.

^c^Adaptive in nature, meaning that participants would only answer questions based on their answers to previous items.

^d^CAD–MDD: Computerized Adaptive Diagnosis for Major Depressive Disorder.

^e^DSM–IV: Diagnostic and Statistical Manual of Mental Disorders (fourth edition).

^f^SCID–I: Structured Clinical Interview for DSM Axis I Disorders.

^g^DSM–IV–TR: DSM–IV (text revision).

^h^Unclear.

^i^Major depressive episode or mania or hypomania.

^j^Panic disorder or generalized anxiety disorder.

^k^Major depressive episode (unspecified).

^l^GAD: generalized anxiety disorder.

^m^WMH–ICS: World Health Organization World Mental Health International College Student.

^n^MINI: Mini-International Neuropsychiatric Interview.

^o^Yes.

^p^PHQ-2: 2-item Patient Health Questionnaire.

^q^GAD-2: 2-item Generalized Anxiety Disorder Scale.

^r^CIDI: Composite International Diagnostic Interview.

^s^MDD, dysthymia, or minor depression.

^t^OCD: obsessive-compulsive disorder.

^u^PTSD: posttraumatic stress disorder.

^v^AUD: alcohol use disorder.

^w^WSQ: Web-Based Screening Questionnaire.

^x^GAD–7: 7-item Generalized Anxiety Disorder Scale.

^y^CES–D: Center for Epidemiological Studies–Depression Scale.

^z^PDSS: Panic Disorder Severity Scale.

^aa^FQ: Fear Questionnaire.

^ab^IES–R: Impact of Event Scale–Revised.

^ac^YBOCS: Yale–Brown Obsessive Compulsive Scale.

^ad^AUDIT: Alcohol Use Disorders Identification Test.

^ae^SID: single-item depression scale.

^af^K10: Kessler Psychological Distress Scale.

^ag^GAD, panic disorder, social phobia, or PTSD.

^ah^GAD–SI: single-item Generalized Anxiety Disorder Scale.

^ai^PHQ–9: 9-item Patient Health Questionnaire.

^aj^EUPD: emotionally unstable personality disorder.

^ak^Also known as borderline personality disorder.

^al^PID–5: Personality Inventory for the DSM–5.

^am^FFM: Five Factor Model questionnaire.

^an^SCID–II–PQ: Structured Clinical Interview for DSM Axis II Disorders Personality Questionnaire.

^ao^SCID–II: Structured Clinical Interview for DSM Axis II Disorders.

^ap^MDD, bipolar depression, bipolar spectrum disorder, GAD, agoraphobia, panic disorder, social phobia, PTSD, or OCD.

^aq^GAD, agoraphobia, panic disorder, social phobia, PTSD, or OCD.

^ar^MDD or bipolar depression.

^as^M-3: My Mood Monitor.

^at^MDD or minor depression.

^au^CAT–DI: Computerized Adaptive Test–Depression Inventory.

^av^No.

^aw^CAT–ANX: Computerized Adaptive Test–Anxiety.

^ax^The authors also used the CAT–DI, but no accuracy data were reported.

^ay^CAT–Psychosis: Computerized Adaptive Test–Psychosis.

^az^MHS: A: Mental Health Screening Tool for Anxiety Disorders.

^ba^SI–Bord: screening instrument for borderline personality disorder.

^bb^SUD: substance use disorder.

^bc^SISQ: single-item screening question.

^bd^Depression (unspecified) or dysthymia.

^be^BN: bulimia nervosa.

^bf^e-PASS: electronic psychological assessment screening system.

^bg^ADIS–IV: Anxiety Disorders Interview Schedule (fourth edition).

^bh^MDI: Major Depression Inventory.

^bi^M–CIDI: Munich–Composite International Diagnostic Interview.

^bj^BD: bipolar disorder.

^bk^ADHD: attention-deficit/hyperactivity disorder.

^bl^CMFC: Connected Mind Fast Check.

^bm^SAM: standardized assessment module.

^bn^SCID–V–RV: Structured Clinical Interview for the DSM–5 Research Version.

^bo^TAPS–1: Tobacco, Alcohol, Prescription Medication, and Other Substance Use scale.

^bp^NIDA: National Institute on Drug Abuse.

^bq^Anxiety disorder (unspecified).

^br^BSA: Brief Scale for Anxiety.

^bs^PI–R: Padua Inventory–Revised.

^bt^PAI: Panic Appraisal Inventory.

^bu^PSWQ: Penn State Worry Questionnaire.

^bv^WDQ: Worry Domains Questionnaire.

^bw^SIAS: Social Interaction and Anxiety Scale.

^bx^SPS: Social Phobia Scale.

^by^AN: anorexia nervosa.

^bz^BED: binge eating disorder.

^ca^EDNOS: eating disorder not otherwise specified.

^cb^EDQ–O: Eating Disorder Questionnaire–Online.

^cc^UBCS: Ultra Brief Checklist for Suicidality.

#### Any Mood or Anxiety Disorder Identification

A total of 1 study (1/28, 4%) targeted the identification of *any* mood or anxiety disorder [28]. To do this, the authors used the My Mood Monitor (M-3) checklist, which is a commercially available test developed by a panel of mental health clinicians and researchers and intended for use in primary care. The tool consists of a total of 27 items focusing on the presence of psychiatric symptoms over the past 2 weeks and covers the following disorders: MDD (7 questions), generalized anxiety disorder (GAD; 2 questions), panic disorder (2 questions), social phobia (1 question), PTSD (4 questions), and OCD (3 questions). In addition, the M-3 inquires about lifetime symptoms of BD (4 questions) and includes a set of 4 functional impairment questions. The authors assessed whether a positive screen on any of the diagnostic categories could be used to identify any mood or anxiety disorder. The sensitivity and specificity of the M-3 were 0.83 and 0.76, respectively.

#### Any Mood Disorder Identification

The study by Ballester et al [21] targeted the identification of *any* mood disorder. To this end, the authors used the World Health Organization World Mental Health International College Student (WMH–ICS) surveys, which are based on existing questionnaires and include a total of 291 questions. These surveys were designed to generate epidemiological data on mental health disorders among college students worldwide. For current mood disorders, the sensitivity and specificity of the WMH–ICS surveys were 0.76 and 0.80, respectively (AUC=0.78). Lifetime/past mood disorders were identified with a sensitivity of 0.95 and a specificity of 0.60 (AUC=0.77). Overall, discrimination ability was fair for both current and lifetime prevalence of mood disorders.

#### Any Anxiety Disorder Identification

A total of 4 studies (4/28, 14%) targeted *any* anxiety disorder [21,25,28,45], resulting in a total of 13 unique tools. The study by Ballester et al [21] used the WMH–ICS surveys, which had a sensitivity of 0.79 and a specificity of 0.89 (AUC=0.84) for current anxiety disorders. Lifetime anxiety disorders were identified with a sensitivity of 0.92 and a specificity of 0.71 (AUC=0.81). Accuracy was good for both current and lifetime prevalence of any anxiety disorder.

Digitized versions of the well-validated 7-item Generalized Anxiety Disorder Scale (GAD–7) and its more succinct versions, the 2-item (GAD–2) and single-item (GAD–SI) scales, were used by Donker et al [25]. For cutoff scores with the highest Youden indexes, the sensitivity and specificity of these tools were 0.36 and 0.78 (GAD–7), 0.47 and 0.72 (GAD–2), and 0.72 and 0.41 (GAD–SI), respectively.

The Brief Scale for Anxiety, Padua Inventory–Revised, Panic Appraisal Inventory, Penn State Worry Questionnaire, Worry Domains Questionnaire, Social Interaction and Anxiety Scale, Social Phobia Scale, and Impact of Event Scale–Revised were used in their digitized versions by Schulte-van Maaren et al [45]. The total number of questions varied from 15 to 21, with excellent discrimination ability (AUC=0.92-0.96). The sensitivity and specificity values for these tools ranged from 0.86 to 0.91 and 0.85 to 0.91, respectively.

Finally, the study by Gaynes et al [28] used the anxiety items of the M-3 (ie, GAD, panic disorder, social phobia, PTSD, and OCD), comprising a total of 12 questions. The sensitivity and specificity of the M-3 were 0.82 and 0.78, respectively.

#### Any Depressive Disorder Identification

Among the 8 studies (8/28, 29%) targeting the recognition of *any* depressive disorder [21,23-25,28,29,38,43], 11 unique digital mental health assessments were used. These comprised a combination of digitized versions of existing questionnaires, including the single-item depression scale, Center for Epidemiological Studies–Depression Scale, and Kessler Psychological Distress Scale as well as the GAD–7, GAD–2, and GAD–SI, with the total number of questions ranging from 1 to 20. For cutoff scores with the highest Youden indexes, the sensitivity and specificity of these tools were 0.87 and 0.51 (single-item depression scale [24]), 0.94 and 0.69 (Center for Epidemiological Studies–Depression Scale [23,24]), 0.71 and 0.77 (Kessler Psychological Distress Scale [24]), 0.94 and 0.37 (GAD–7 [25]), 0.61 and 0.75 (GAD–2 [25]), and 0.82 and 0.43 (GAD–SI [25]), respectively.

In addition, tools based on existing questionnaires included the WMH–ICS–Major Depressive Episode survey (current: sensitivity=0.93, specificity=0.83, AUC=0.88; lifetime: sensitivity=0.96, specificity=0.65, AUC=0.80), which demonstrated good accuracy [21], and the 2 MDD items of the 15-item Web-Based Screening Questionnaire (WSQ; sensitivity=0.85 [23] and 0.58 [38], specificity=0.59 [23] and 0.94 [37]), which showed fair to good discrimination ability (AUC=0.72 [23] and 0.83 [38]). The WSQ is based on an existing questionnaire, the Mini-International Neuropsychiatric Interview, and the Alcohol Use Disorders Identification Test and can be used to assess depression, GAD, panic disorder, panic disorder with agoraphobia, agoraphobia, specific phobia, social phobia, PTSD, OCD, alcohol abuse and dependence, and suicide.

Furthermore, 1 study (1/28, 4%) [28] used the 7 MDD questions of the M-3 (sensitivity=0.84, specificity=0.80), whereas another study (1/28, 4%) [29] used the Computerized Adaptive Test–Depression Inventory (CAT–DI), which includes a total of 389 items and comprises one of the modules of the commercially available Computerized Adaptive Test–Mental Health (CAT–MH). These modules are based on existing questionnaires, DSM–IV criteria, and an expert panel. Notably, the tests can be fully integrated into routine care and are adaptive in nature, meaning that participants only answer questions based on their answers to previous items. The accuracy of the CAT–DI varied depending on the comparison group (nonpsychiatric comparator: sensitivity=0.90, specificity=0.88; psychiatric comparator: sensitivity=0.90, specificity=0.64). Finally, the study by Rogers et al [43] used the Connected Mind Fast Check (CMFC), which was developed by an expert panel that included psychologists. The tool screens and assesses for several psychiatric disorders using initial screeners and standardized assessment modules (SAMs). The number of questions ranges from 1 to 2 for the initial screeners, resulting in a total of 8 screening questions, and between 11 and 27 for the SAMs. The SAMs are adaptive in nature, meaning that individuals only answer questions based on their answers to previous items. Notably, the CMFC is eligible for reimbursement for primary care practices in the United States. In terms of diagnostic accuracy, the sensitivity and specificity of the CMFC initial screener were 0.94 and 0.65, respectively. In contrast, the SAM had a sensitivity of 0.45 and a specificity of 0.93. Importantly, when reviewing the decision rules of the CMFC SAM, the capability of the tool to detect a major depressive episode increased to 0.73 (sensitivity), whereas the specificity remained largely unchanged (0.92).

#### Generalized Anxiety Disorder Identification

A total of 12 studies (12/28, 43%) focused on the identification of GAD [21-23,25,31,32,34,35,38-40,43], comprising a total of 9 unique tools. The most popular assessments were the digitized version of the GAD–7, with sensitivity and specificity values ranging from 0.75 to 0.87 and 0.55 to 0.78, respectively [23,25,34,39]. Discrimination ability for digitized versions of the GAD–7 ranged from poor to good (AUC=0.65-0.86). Diagnostic validity for GAD identification was also assessed for the Computerized Adaptive Test–Anxiety (CAT–ANX), which comprises one of the modules of the CAT–MH. The sensitivity and specificity of the CAT–ANX varied depending on the sample type (entire sample: sensitivity=0.89, specificity=0.77; nonpsychiatric comparator: sensitivity=0.86, specificity=0.86 [31]). In addition, the study by Graham et al [32] demonstrated that the CAT–ANX was excellent at discriminating individuals with GAD from those without the condition (AUC=0.93).

Other tools included the digitized versions of the GAD–2, which was used by both Cano-Vindel et al [22] (sensitivity=0.77, specificity=0.80) and Donker et al [25] (sensitivity=0.83, specificity=0.61, AUC=0.76), as well as the GAD–SI (sensitivity=0.70, specificity=0.76 [25]), which showed fair discrimination ability (AUC=0.78). The GAD survey of the WMH–ICS demonstrated good to excellent accuracy (current: sensitivity=1.00, specificity=0.86, AUC=0.93; lifetime: sensitivity=0.97, specificity=0.79, AUC=0.88 [21]). In addition, the GAD item of the WSQ was used across 2 studies, with discrimination ability ranging from fair to good (Donker et al [23]: sensitivity=0.93, specificity=0.45, AUC=0.78; Meuldijk et al [38]: sensitivity=0.66, specificity=0.90, AUC=0.89).

GAD was assessed using the GAD module of the electronic psychological assessment screening system (e-PASS), which is based on the DSM–IV text revision criteria (sensitivity=0.78, specificity=0.68 [40]). The e-PASS assesses a total of 21 disorders; includes >540 questions; and is adaptive in nature, meaning that participants only answer questions based on their answers to previous items. It also includes a number of sociodemographic questions. The e-PASS is funded by the Australian Government Department of Health and Ageing and is available on the web for free. Upon completion, recommendations on what to do next (eg, referral to another service) are provided to individuals. If needed, the e-PASS provides e-therapist support via email, video, or chat. This is intended to help guide users and is not a replacement for face-to-face care.

Furthermore, GAD was also assessed using the Mental Health Screening Tool for Anxiety Disorders [35], which demonstrated excellent diagnostic accuracy (sensitivity=0.98, specificity=0.80, AUC=0.95). The tool comprises 11 questions based on existing questionnaires and diagnostic criteria, focus group interviews with patients with GAD, and an expert panel. Finally, the study by Rogers et al [43] used the CMFC. The initial screener had a sensitivity of 0.93 and a specificity of 0.63, whereas the SAM resulted in a sensitivity and specificity of 0.73 and 0.89, respectively. The sensitivity of the SAM increased to 0.90 when reviewing the module’s decision rules, with the specificity remaining largely unchanged (0.86).

#### Panic Disorder Identification

Among the 7 studies (7/28, 25%) targeting the recognition of panic disorder [21,23,25,38,40,42,43], 8 unique digital mental health assessment tools were used. The most popular tool for panic disorder was the panic disorder item of the WSQ, which was used by Donker et al [23] (sensitivity=0.90, specificity=0.44, AUC=0.76), Meuldijk et al [38] (sensitivity=0.81, specificity=0.95, AUC=0.98), and Oromendia et al [42] (sensitivity=0.81, specificity=0.80, AUC=0.82). Other tools used included the digitized versions of the GAD–7 (sensitivity=0.88, specificity=0.37, AUC=0.62 [25]), GAD–2 (sensitivity=0.38, specificity=0.83, AUC=0.64 [25]), and GAD–SI (sensitivity=0.88, specificity=0.39, AUC=0.65 [25]) as well as the self-reported version of the Panic Disorder Severity Scale (AUC=0.70 [23]). In addition, the panic disorder questions of the e-PASS (sensitivity=0.71, specificity=0.91 [40]) and WMH–ICS (current: sensitivity=0.45, specificity=0.98, AUC=0.71; lifetime: sensitivity=0.71, specificity=0.83, AUC=0.77 [21]) were also used to assess the condition. Finally, the study by Rogers et al [43] used the CMFC. The initial screener had a sensitivity of 0.79 and a specificity of 0.52, whereas the SAM resulted in a sensitivity and specificity of 0.32 and 0.76, respectively.

#### Social Phobia Identification

A total of 5 studies (5/28, 18%) focused on the recognition of social phobia [23,25,38,40,43], comprising a total of 7 unique digital mental health assessment tools. The social phobia items of the WSQ were used across 2 studies (2/28, 7%; sensitivity=0.72, specificity=0.73, AUC=0.72 [23]; sensitivity=0.79, specificity=0.93, AUC=0.95 [38]). The accuracy of the GAD–7 (sensitivity=0.38, specificity=0.77 [25]) and GAD–2 (sensitivity=0.46, specificity=0.70 [25]) was also evaluated, and both presented AUCs <0.60, which is generally regarded as a fail. Other tools included the GAD–SI (sensitivity=0.69, specificity=0.39, AUC=0.76 [25]), the Fear Questionnaire (FQ; AUC=0.82 [23]), and the social phobia items of the e-PASS (sensitivity=0.60, specificity=0.90 [40]). In addition, the study by Rogers et al [43] used the CMFC. The initial screener had a sensitivity of 0.92 and a specificity of 0.53, whereas the SAM resulted in a sensitivity and specificity of 0.42 and 0.75, respectively.

#### PTSD Identification

A total of 5 studies (5/28, 18%) targeted PTSD [23,25,28,38,40], resulting in 7 unique digital mental health assessment tools with accuracies ranging from poor to good. The PTSD items of the WSQ were used by Donker et al [23] (sensitivity=0.83, specificity=0.47, AUC=0.65) and Meuldijk et al [38] (sensitivity=0.79, specificity=0.52, AUC=0.86). Other tools included the digitized versions of the GAD–7 (sensitivity=0.75, specificity=0.77, AUC=0.76 [25]), GAD–2 (sensitivity=0.88, specificity=0.71, AUC=0.74 [25]), GAD–SI (sensitivity=0.63, specificity=0.69, AUC=0.69 [25]), and Impact of Event Scale (AUC=0.82 [23]), which includes a total of 15 items. In addition, the PTSD items of the e-PASS (sensitivity=0.75, specificity=0.92 [40]) and M-3 (sensitivity=0.88, specificity=0.70 [28]) were used to assess for the presence of the disorder.

#### OCD Identification

OCD was assessed using 3 unique digital mental health assessments across 3 separate studies (3/28, 11%) [23,38,40]. The OCD item of the WSQ was used in 2 studies (2/28, 7%), with a sensitivity and specificity of 0.80 and 0.69 [23] and 0.67 and 0.91 [38], respectively, and a good discrimination ability in both studies (AUC=0.81 [23], AUC=0.82 [38]). The remaining 2 tools included the OCD items of the e-PASS (sensitivity=0.75, specificity=0.92 [40]) and the digitized version of the Yale–Brown Obsessive Compulsive Scale, which comprises a total of 10 questions and showed good accuracy (AUC=0.86 [23]).

#### Agoraphobia Identification

A total of 2 studies (2/28, 7%) targeted the identification of agoraphobia [23,38] with good accuracy. In both studies, the authors used the agoraphobia item of the WSQ (sensitivity=1.00, specificity=0.63, AUC=0.81 [23]; sensitivity=0.81, specificity=0.95, AUC=0.80 [38]). Donker et al [23] also used the digitized version of the FQ, which includes 5 questions to assess the condition (AUC=0.81).

#### MDD Identification

Among the 8 studies (8/28, 29%) focusing on MDD [20,22,26,29,30,32,40,41], a total of 6 digital mental health assessment tools were used. The most widely used tool was the Computerized Adaptive Diagnosis for MDD (CAD–MDD), which comprises one of the modules of the CAT–MH and consists of a total of 389 questions. The accuracy of the CAD–MDD varied across studies and sample types (sensitivity=0.77-0.96, specificity=0.64-1.00 [20,30,32]). The CAT–DI was used by Gibbons et al [29], with a sensitivity of 0.82 and a specificity of 0.85. The MDD module of the e-PASS was used by Nguyen et al [40] (sensitivity=0.86, specificity=0.79), whereas 2 studies (2/28, 7%) used the digitized versions of the PHQ–9 with good accuracy (sensitivity=0.89, specificity=0.79, AUC=0.90 [26]) and the 2-item Patient Health Questionnaire (sensitivity=0.78, specificity=0.73 [22]). Finally, the study by Nielsen et al [41] used the Major Depression Inventory, which is a digital version of an existing questionnaire and includes 13 questions, resulting in poor accuracy (sensitivity=0.62, specificity=0.63, AUC=0.66).

#### BD or Bipolar Spectrum Disorder Identification

In total, 1 study (1/28, 4%) targeted lifetime bipolar spectrum disorder [28] using the 4 BD items of the M-3, which had a sensitivity of 0.88 and a specificity of 0.70. In addition, the study by Rogers et al [43] used the CMFC to detect BD in individuals who met the criteria for a major depressive episode. The initial screener had a sensitivity of 0.63 and a specificity of 0.79, whereas the SAM resulted in a sensitivity and specificity of 0.50 and 0.97, respectively.

#### ADHD Identification

A total of 1 study (1/28, 4%) assessed for ADHD [43] using the CMFC. The initial screener resulted in a sensitivity and specificity of 0.94 and 0.61, respectively, whereas the SAM had a sensitivity of 0.69 and a specificity of 0.86.

#### AUD and SUD Identification

A total of 5 studies (5/28, 18%) targeted the identification of AUD [23,37,38,40,44] using a total of 5 distinct digital mental health assessment tools with fair to good accuracy. The alcohol items of the WSQ were used by both Donker et al [23] (sensitivity=0.83, specificity=0.72, AUC=0.77) and Meuldijk et al [38] (sensitivity=0.56, specificity=0.92, AUC=0.82). Other tools included the alcohol module of the e-PASS (sensitivity=0.42, specificity=1.00 [40]) as well as the digitized versions of the single-item screening question (SISQ) for AUD (SISQ–alcohol; sensitivity=0.87, specificity=0.74, AUC=0.80 [37]); Tobacco, Alcohol, Prescription Medication, and Other Substance Use tool (sensitivity=0.97, specificity=0.99 [44]); and Alcohol Use Disorders Identification Test (AUC=0.75 [28]).

A total of 2 studies (2/28, 7%) focused on SUD. The study by McNeely et al [37] used the SISQ–drugs, which had a sensitivity of 0.85 and a specificity of 0.89 (AUC=0.87). The study by Rogers et al [43] used the CMFC. The initial screener had a sensitivity of 0.80 and a specificity of 0.92, whereas the SAM resulted in a sensitivity and specificity of 0.67 and 0.96, respectively.

#### Eating Disorders Identification

Regarding eating disorders, 1 study (1/28, 4%) [46] focused on anorexia nervosa and bulimia nervosa (BN) as well as binge eating disorder and eating disorder otherwise not specified using the Eating Disorder Questionnaire–Online (EDQ–O), which is based on the Mini-International Neuropsychiatric Interview–Plus and DSM–IV text revision criteria and comprises a total of 26 questions. The accuracy of the EDQ–O for the recognition of these conditions ranged from fair to good (anorexia nervosa: sensitivity=0.44, specificity=1.00, AUC=0.72; BN: sensitivity=0.78, specificity=0.88, AUC=0.83; binge eating disorder: sensitivity=0.66, specificity=0.98, AUC=0.82; eating disorder otherwise not specified: sensitivity=0.87, specificity=0.72, AUC=0.79). An additional study (1/28, 4%) [40] targeted BN using the bulimia module of the e-PASS, which had a sensitivity and specificity of 0.50 and 0.97, respectively.

#### Emotionally Unstable Personality Disorder Identification

When considering personality disorders, 2 studies (2/28, 7%) targeted emotionally unstable personality disorder (EUPD) [27,36], also known as borderline personality disorder. Fowler et al [27] used digitized versions of the Five Factor Model, with a sensitivity of 0.70 and a specificity of 0.62 for the neuroticism and agreeableness composites and a sensitivity and specificity of 0.71 and 0.62, respectively, for the neuroticism, agreeableness, and conscientiousness composites. Both combinations of composites had fair accuracy (AUC=0.72 and 0.73, respectively). The authors also used the self-report Structured Clinical Interview for DSM Axis II Disorders Personality Questionnaire, which had a sensitivity and specificity of 0.78 and 0.80, respectively, and good discrimination ability (AUC=0.86), and the Personality Inventory for the DSM–5 (sensitivity=0.81, specificity=0.76), which also showed good accuracy (AUC=0.87). Lohanan et al [36] used the screening instrument for borderline personality disorder, which is based on the Structured Clinical Interview for DSM Axis II Disorders and includes a total of 5 items. The sensitivity of the screening instrument for borderline personality disorder was 0.56, whereas the specificity was 0.92 with good accuracy (AUC=0.83).

#### Psychosis Identification

In total, 1 study (1/28, 4%) targeted psychosis [33] using the Computerized Adaptive Test–Psychosis (CAT–Psychosis), which is one of the tests available in the CAT–MH. The accuracy of the CAT–Psychosis was good (entire sample: AUC=0.85; including only those who had received the Structured Clinical Interview for DSM Axis I Disorders: AUC=0.80).

#### Suicidality Identification

A total of 2 studies (2/28, 7%) examined suicidality. The first study [43] used the CMFC, with the accuracy of the initial screener varying depending on the criteria examined (thoughts of own death: sensitivity=0.75, specificity=0.89; suicidal ideation: sensitivity=0.75, specificity=0.84; specific plan: sensitivity=1.00, specificity=0.80). The second study [47] used the Ultra Brief Checklist for Suicidality, which had a sensitivity of 0.91 and a specificity of 0.85 for the cutoff score with the highest Youden index.

### Risk of Bias and Applicability Assessment

The evaluation of risk of bias and applicability for all 28 studies was conducted using the amended QUADAS–2 tool [17]. The results are summarized in Table 3, with scores for each signaling question available upon request. This assessment revealed a high risk of bias in most of the considered studies. For instance, with regard to patient selection, 12 studies (12/28, 43%) [20,24,29-33,38,40,42,45,47] had high risk of bias, primarily because of issues with enrollment and a failure to avoid a case–control sample, which may not fully reflect real-world patient populations. A total of 9 studies (9/28, 32%) [21-23,25,34,36,39,44,46] did not provide enough information regarding their sample and sampling procedures. Similarly, risk of bias was an issue when considering index test administration, with 10 studies (10/28, 36%) [21,24-28,35,36,40,47] showing high risk of bias, which was primarily due to the studies not using a prespecified threshold. A total of 13 studies (13/28, 46%) [20,22,29-31,33,34,38,42-46] failed to provide enough information regarding the index test administration. This was particularly with regard to whether the results were interpreted without knowledge of the reference standard. In total, 1 study (1/28, 4%) [29] showed high risk of bias when considering the reference standard, with the results interpreted with knowledge of the results of the index test, whereas 14 studies (14/28, 50%) [20,22,24,27,30,31,33,34,36-38,42,44,45] did not provide sufficient information regarding the interpretation of the reference standard. Finally, flow and timing were also a consideration, with 4 studies (4/28, 14%) showing high risk of bias. In this regard, Guinart et al [33] did not re-administer the reference standard to patients who had received a diagnostic interview within the 12 months before taking part in the study, and the studies by Gibbons et al [29-31] included nonpsychiatric controls in the analyses who appeared not to have received the reference standard. A total of 11 studies (11/28, 39%) [20,22-25,34-36,38,39,45] did not provide enough information regarding the timing between the index test and reference standard.

In terms of applicability, given our review question and strict inclusion and exclusion criteria, all the included studies were judged to have low applicability concerns.

**Table 3:** Results of the amended quality assessment of the included studies.

Study	Risk of bias					Applicability concerns		
	Patient selection	Index test	Reference standard	Flow and timing	Patient selection		Index test	Reference standard
Achtyes et al [20]	 ^a^	?^b^	?	?	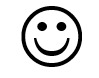 ^c^		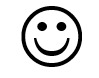	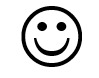
Ballester et al [21]	?		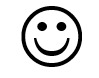	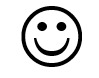	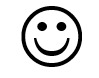		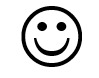	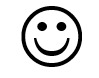
Cano-Vindel et al [22]	?	?	?	?	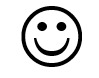		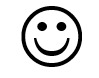	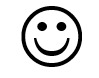
Donker et al [23]	?	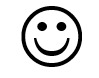	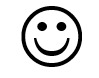	?	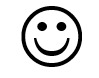		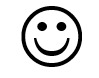	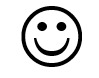
Donker et al [24]			?	?	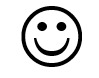		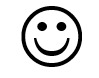	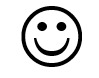
Donker et al [25]	?		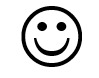	?	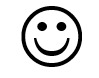		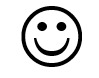	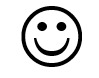
Du et al [26]	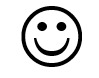		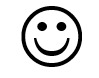	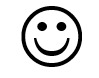	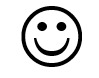		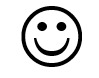	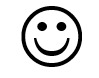
Fowler et al [27]	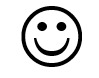		?	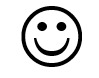	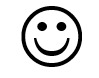		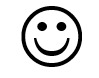	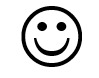
Gaynes et al [28]	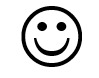		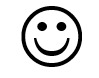	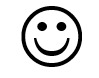	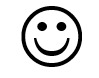		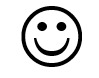	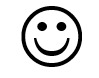
Gibbons et al [29]		?			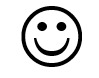		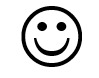	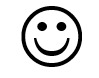
Gibbons et al [30]		?	?		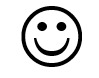		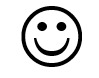	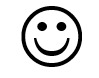
Gibbons et al [31]		?	?		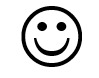		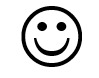	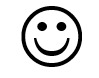
Graham et al [32]		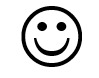	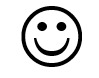	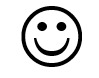	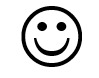		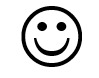	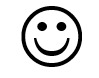
Guinart et al [33]		?	?		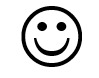		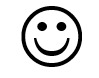	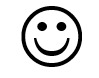
Kertz et al [34]	?	?	?	?	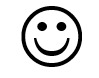		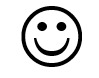	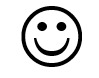
Kim et al [35]	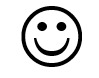		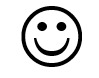	?	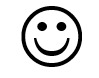		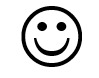	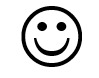
Lohanan et al [36]	?		?	?	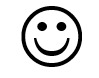		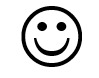	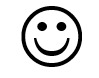
McNeely et al [37]	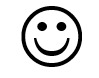	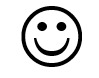	?	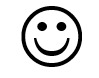	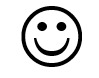		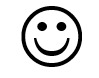	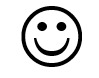
Meuldijk et al [38]		?	?	?	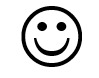		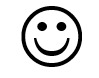	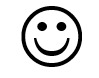
Munoz-Navarro et al [39]	?	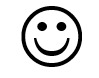	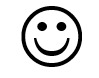	?	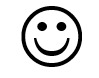		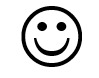	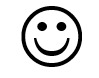
Nguyen et al [40]			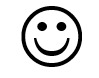	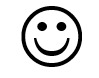	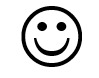		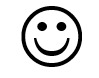	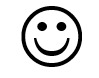
Nielsen et al [41]	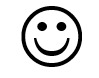	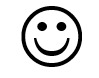	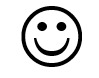	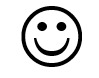	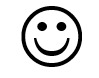		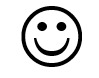	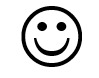
Oromendia et al [42]		?	?	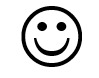	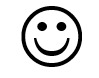		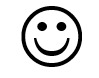	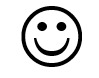
Rogers et al [43]	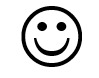	?	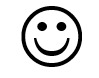	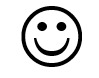	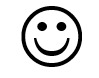		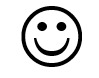	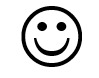
Sanchez et al [44]	?	?	?	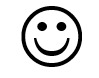	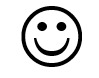		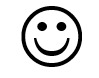	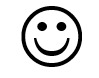
Schulte-van Maaren et al [45]		?	?	?	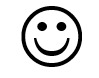		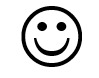	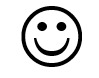
Ter Huurne et al [46]	?	?	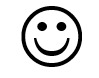	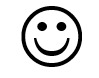	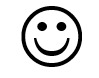		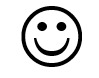	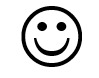
Yoon et al [47]			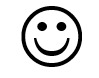	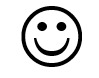	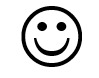		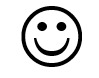	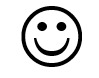

^a^High risk.

^b^Unclear risk.

^c^Low risk.

## Discussion

### Overview

This systematic review set out to explore the current state and validity of question-and-answer–based digital mental health assessment tools targeting a wide range of mental health conditions. We believe that the findings of this review will provide health care professionals and researchers with a deeper understanding of the use of digital technologies for the screening and diagnosing of mental health conditions in adulthood, as well as of the challenges that remain and opportunities for the development of innovative digital mental health assessment tools moving forward.

#### Implications for Health Care Professionals

The digitization of existing pen-and-paper questionnaires and scales routinely used for mental health screening and assessment can offer various benefits, such as minimal delivery costs, efficient data collection, and increased convenience. For health care providers looking to digitize the use of existing pen-and-paper questionnaires in their clinical practice, the included studies report on 26 unique tools. Critically, most of these tools were designed to target a single condition rather than being comprehensive assessments of psychopathology, with most including <45 questions. Thus, a combination of these tools should be considered if a comprehensive mental health assessment is preferred.

Alternatively, tools targeting several conditions, such as the M-3 [28], WHM–ICS surveys [21], WSQ [23,38,42], e-PASS [40], and CMFC [43], may represent more attractive options for mental health screening in primary care settings and the first stages of triage. Notably, only the e-PASS includes sociodemographic questions, providing valuable information on factors that are known to be correlated with mental health concerns [48]. In addition, the e-PASS is adaptive in nature, meaning that participants only answer questions based on their answers to previous items, which can ensure that assessment completion is more time-efficient and only relevant symptom data are collected. Adaptive testing was also offered by the CMFC, which is eligible for reimbursement for primary care practices in the United States, as well as by the CAD–MDD, CAT–DI, CAT–ANX, and CAT–Psychosis, which are commercially available.

Overall, the intended settings of use should be carefully considered by health care professionals interested in implementing digital mental health assessment tools in their clinics. Similarly, the importance of accuracy measures in choosing relevant digital tools cannot be overstated. This systematic review revealed mixed findings regarding the validity of the included digital technologies, with accuracy values varying significantly between and within conditions and instruments as well as across different samples. Sensitivity and specificity values ranged from 0.32 to 1.00 and 0.37 to 1.00, respectively, and AUCs ranged from poor (0.57) to excellent (0.98).

Specifically, the GAD–7 and its more succinct versions, which represent the most frequently used instruments, generally demonstrated poor to fair discriminatory performance across a range of anxiety disorders [23,25,34]. An exception was the study by Munoz-Navarro et al [39], where the GAD–7 showed good accuracy in identifying GAD. The digitized versions of existing pen-and-paper questionnaires used by Schulte-van Maaren et al [45] with the aim of identifying any anxiety disorder had excellent accuracy, whereas digitized versions of the FQ, Impact of Event Scale–Revised, and Yale–Brown Obsessive Compulsive Scale demonstrated good discriminatory performance for a variety of anxiety disorders [23]. Regarding digitized versions of existing pen-and-paper questionnaires targeting conditions other than anxiety, the PHQ–9 demonstrated excellent accuracy for MDD [26], whereas the 2-item Patient Health Questionnaire was only fair [22], and the Major Depression Inventory demonstrated poor performance in identifying the condition [41]. SISQs for both AUD and SUD had good accuracy [37], whereas tools assessing for EUPD demonstrated fair to good discriminatory performance [27]. Importantly, although the screening or diagnostic accuracy of these digitized versions of existing pen-and-paper questionnaires appeared to vary significantly across studies, previous systematic reviews have generally revealed good interformat reliability between digital and paper versions, suggesting that these are comparable [49,50]. Therefore, differences in screening or diagnostic accuracy are likely to be due to study effects or methodological issues rather than the tools used being unreliable. Moving forward, there is a need for carefully designed, high-quality studies to further validate and assess the clinical utility of digitized versions of pen-and-paper questionnaires. This will help guide clinicians toward meaningful technologies.

Regarding tools that were not a digitized version of existing pen-and-paper questionnaires and instead gathered questions designed ex novo by mental health experts based on existing diagnostic tools and criteria, the WMH–ICS surveys demonstrated good to excellent accuracy for the identification of any anxiety and depressive disorder as well as GAD [21]. However, the accuracy of the WMH–ICS surveys was fair for any mood disorder and panic disorder [21]. In contrast, the Mental Health Screening Tool for Anxiety Disorders [35] and Tobacco, Alcohol, Prescription Medication, and Other Substance Use scale [44] were excellent at identifying GAD and AUD, respectively. Similarly, the SI-Bord demonstrated good accuracy for EUPD [36], whereas the Ultra Brief Checklist for Suicidality had a sensitivity and specificity of 0.91 and 0.85, respectively, for suicidality [47]. Regarding eating disorders, the EDQ–O presented fair to good discriminatory performance [46].

In addition, the accuracy of the WSQ varied from poor to excellent depending on the condition of interest and study [23,38,42]. Similarly, the clinical utility of the e-PASS varied considerably across conditions, with sensitivity and specificity values ranging from 0.42 to 0.86 and 0.68 to 1.00, respectively [40]. The accuracy of the CMFC also varied across conditions, with sensitivity and specificity values ranging from 0.63 to 1.00 and 0.61 to 0.92 (initial screener) and from 0.32 to 0.75 and 0.90 to 0.97 (SAMs), respectively [43]. Furthermore, the accuracy of the CAD–MDD, CAT–DI, CAT–ANX, and CAT–Psychosis varied across studies and depending on the comparison group (eg, nonpsychiatric comparator vs psychiatric comparator) [20,29-33]. Of these, the CAD–MDD was conceptualized and developed as a screening tool for depression in primary care, whereas the CAT–DI and CAT–ANX are better suited for assessing depression and anxiety severity, respectively [30,32]. Taken together in the form of the CAT–MH, these adaptive assessments could provide a valuable screening and assessment tool for depression and anxiety [32]. The CAT–Psychosis served as a discriminating tool for the presence of psychosis and as an assessment tool for symptom severity, thereby being well-placed in secondary care for psychosis screening and follow-up assessments. Finally, the accuracy of the M-3 varied across conditions, with sensitivity and specificity values ranging from 0.82 to 0.88 and 0.70 to 0.80, respectively [28].

Overall, the utility of the tools included in this review will strongly depend on clinical needs. For screening purposes, tools that have high sensitivity and that can be easily completed by patients are to be prioritized. In contrast, tools with high specificity perform well for diagnostic purposes in symptomatic patient populations. The implementation of digital mental health assessments in common practice workflows will likely require pilot-testing to tailor the tool to case-specific needs.

#### Recommendations for Research

In addition to reporting on digital mental health assessments’ features and accuracy, this systematic review highlights tool development and study design considerations that may inform future research aims. Although the diagnosis of GAD, any depressive disorder, and MDD was investigated in several studies, fewer eligible studies were found for specific anxiety disorders, such as panic disorder and social phobia, as well as AUD. Notably, very few studies targeted the identification of BD, ADHD, SUD, psychosis, and suicidality. Thus, there remain opportunities for the development of more comprehensive digital diagnostic tools. Indeed, digital technologies have the capacity to collect a vast range of key sociodemographic and symptom data. Undeniably, by moving away from brief symptom count checklists such as the GAD–7 and PHQ–9, digital technologies can offer avenues toward a dimensional view of psychopathology, providing valuable information on the co-occurrence of symptoms and diagnoses. Indeed, digital technologies, including adaptive or nonlinear questionnaires where patients are required to answer questions based on previous answers, have the capacity to further streamline and personalize the collection of cross-disorder symptom data. Although outside the scope of this systematic review, combining clinical information with biomarker profiling strategies may allow clinicians and researchers to further shift the focus from categorical constructs to a dimensional approach to psychopathology. For instance, the combination of symptom data and serum analytes has been shown to predict the development of future depressive episodes in individuals presenting with social anxiety [51] and panic disorder [52]. In addition, combining digital symptom-based data with dried blood spot samples shows some promise as a noninvasive and cost-effective diagnostic test for both MDD [53] and BD [54], but research in this area remains largely unexplored.

In addition to suggesting opportunities for future research, this systematic review raises considerations of methodology and research reporting practices. Indeed, researchers and digital mental health innovators should pursue carefully designed, high-quality studies to validate and assess the clinical utility of their diagnostic tools. Of note, the study by Nielsen et al [41] stood out for their comprehensively written methods and well-designed study. For the remaining studies, risk of bias was a concern despite our amended and less stringent QUADAS–2 measures. This was often due to missing information regarding participant sampling procedures, the administration and interpretation of the index test and reference standard, and timing. Inevitably, the nondisclosure of methodological information can hinder the assessment of bias in current and future systematic review exercises aimed at determining the clinical utility of digital mental health assessments. In addition, missing information can prevent replicability studies from validating the findings. Moving forward, the QUADAS–2 measures could be used by researchers and peer reviewers as a checklist for study procedures that should be clearly reported in study methods in addition to complying with relevant guidelines such as the Standards for Reporting of Diagnostic Accuracy Studies [55]. In particular, careful consideration should be given to patient selection, the index test, the reference standard, and flow and timing. For instance, moving away from a case–control study design, digital mental health care researchers should consider evaluating digital mental health assessment tools within the intended context. This would allow for the appraisal of diagnostic technologies in real-world patient populations, thereby facilitating interoperability and guiding health care professionals toward clinically meaningful technologies.

### Strengths and Limitations

To our knowledge, this is the first systematic review to assess the validity of question-and-answer–based digital mental health assessment tools targeting a wide range of mental health conditions. However, despite our comprehensive and carefully designed search strategies as well as the inclusion of any study design and language, it is possible that some relevant studies may have been missed. Furthermore, given the focus of this review where only digital tools that were exclusively question-and-answer–based were included, diagnostic technologies that collect passive data (eg, activity rhythms, sleep quality, sentiment, and language patterns) or a combination of active and passive data were not evaluated, with further research in this area being required.

### Conclusions

The findings of this systematic review revealed that the field of digital mental health assessment tools is still in its early stages. Indeed, most of the included studies used digitized versions of existing pen-and-paper questionnaires as opposed to more sophisticated and comprehensive digital diagnostic technologies that can be easily integrated into routine clinical care. Furthermore, our review revealed mixed findings regarding the accuracy of the included digital technologies, which varied significantly between and within conditions as well as across different samples. In addition, risk of bias was a concern with the included studies. This comprehensive systematic review has important implications for the development and implementation of digital mental health assessments. Namely, there exist opportunities for further innovation in the field of digital diagnostic technologies for mental health. Importantly, carefully designed, high-quality studies are essential to validate the clinical utility of these technologies. Finally, evaluating these tools within the intended context is likely to facilitate interoperability and help guide clinicians toward meaningful technologies.

## Appendix

Multimedia Appendix 1 Search strategies.

Multimedia Appendix 2 Checklist summary of the mental health disorders investigated in the included studies.

Multimedia Appendix 3 Diagnostic accuracy per index test separated by condition of interest.

## Abbreviations

ADHD: attention-deficit/hyperactivity disorder
AUC: area under the receiver operating characteristic curve
AUD: alcohol use disorder
BD: bipolar disorder
BN: bulimia nervosa
CAD–MDD: Computerized Adaptive Diagnosis for Major Depressive Disorder
CAT–ANX: Computerized Adaptive Test–Anxiety
CAT–DI: Computerized Adaptive Test–Depression Inventory
CAT–MH: Computerized Adaptive Test–Mental Health
CAT–Psychosis: Computerized Adaptive Test–Psychosis
CMFC: Connected Mind Fast Check
DSM: Diagnostic and Statistical Manual of Mental Disorders
EDQ–O: Eating Disorder Questionnaire–Online
e-PASS: electronic psychological assessment screening system
EUPD: emotionally unstable personality disorder
FQ: Fear Questionnaire
GAD: generalized anxiety disorder
GAD–2: 2-item Generalized Anxiety Disorder Scale
GAD–7: 7-item Generalized Anxiety Disorder Scale
GAD–SI: single-item Generalized Anxiety Disorder Scale
ICD: International Statistical Classification of Diseases and Related Health Problems
M-3: My Mood Monitor
MDD: major depressive disorder
OCD: obsessive-compulsive disorder
PHQ–9: 9-item Patient Health Questionnaire
PRISMA: Preferred Reporting Items for Systematic Reviews and Meta-Analyses
PTSD: posttraumatic stress disorder
QUADAS–2: Quality Assessment of Diagnostic Accuracy Studies 2
SAM: standardized assessment module
SISQ: single-item screening question
SUD: substance use disorder
WMH–ICS: World Health Organization World Mental Health International College Student
WSQ: Web-Based Screening Questionnaire
