# Supplementary material for: The Current State and Validity of Digital Assessment Tools for Psychiatry: Systematic Review
Source: JMIR Ment Health. 2022 Mar 30;9(3):e32824. doi: 10.2196/32824 (PMC9008525; doi:10.2196/32824)
Supplement: Multimedia Appendix 1 [file mental_v9i3e32824_app1.docx]

***Multimedia Appendix 1***

**Search strategies:**

***MEDLINE via Ovid*** *(completed October 12^th^, 2021)*

1. ((assess* or diagnostic* or "mood diar*" or PHQ or "PHQ-9" or GAD or "GAD-7" or questionnaire* or screen* or tool* or test* or "computeri#ed adaptive test for mental health" or "CAT-MH" or "e-PASS or WSQ or TAPS or Nview or ada or doctorlink or clinicom) adj5 (accura* or sensitiv* or specific* or "receiver operating characteristic*" or ROC or "area under the curve" or AUC or AUROC or "positive predictive value" or PPV or "negative predictive value" or NPV or precision or recall or "true positive rate" or TPR or "true negative rate" or TNR or valid* or “agreement rate”)).ti,ab.
2. exp "Surveys and Questionnaires"/ and exp "Sensitivity and Specificity"/
3. 1 or 2
4. (app or apps or application* or chatbot* or computer* or "conversational agent*" or device* or digital or "e-health" or ehealth or "e-mental health" or "emental health" or electronic or internet or mhealth or "m-health" or mobile* or online or phone* or smartphone* or “smart-phone*” or cellphone* or “cell-phone*” or telehealth or telemedicine or "text messag*" or web or software or algorithm* or tablet* or PC or PCs).ti,ab.
5. Mobile Applications/ or exp Computers/ or Telemedicine/ or exp Cell Phone/ or exp Internet/
6. 4 or 5
7. ((mood or affective or anxiety or panic or eating or "obsessive compulsive" or "attention deficit hyperactivity" or stress or adjustment or personality) adj disorder*).ti,ab.
8. (depress* or dysthymi* or "MDD" or bipolar or "social phobia*" or ADHD or autism or ASD or insomnia or "anorexia nervosa" or "bulimia nervosa" or OCD or schizophrenia or psychosis or PTSD or BPD or EUPD or "self harm" or "self-harm" or suicid*).ti,ab.
9. (mental* adj (health or ill* or disorder*)).ti,ab.
10. ((alcohol* or drug* or substance*) adj (abuse or addict*)).ti,ab.
11. mental health/ or exp mental disorders/
12. 7 or 8 or 9 or 10 or 11
13. 3 and 6 and 12
14. limit 13 to yr="2005 -Current"

***Embase via Ovid*** *(completed October 12^th^, 2021)*

1. ((assess* or diagnostic* or "mood diar*" or PHQ or "PHQ-9" or GAD or "GAD-7" or questionnaire* or screen* or tool* or test* or "computeri#ed adaptive test for mental health" or "CAT-MH" or "e-PASS or WSQ or TAPS or Nview or ada or doctorlink or clinicom) adj5 (accura* or sensitiv* or specific* or "receiver operating characteristic*" or ROC or "area under the curve" or AUC or AUROC or "positive predictive value" or PPV or "negative predictive value" or NPV or precision or recall or "true positive rate" or TPR or "true negative rate" or TNR or valid* or “agreement rate”)).ti,ab.
2. exp questionnaire/ and (receiver operating characteristic/ or "sensitivity and specificity"/ or validity/ or accuracy/)
3. 1 or 2
4. (app or apps or application* or chatbot* or computer* or "conversational agent*" or device* or digital or "e-health" or ehealth or "e-mental health" or "emental health" or electronic or internet or mhealth or "m-health" or mobile* or online or phone* or smartphone* or “smart-phone*” or cellphone* or “cell-phone*” or telehealth or telemedicine or "text messag*" or web or software or algorithm* or tablet* or PC or PCs).ti,ab.
5. exp mobile application/ or exp computer/ or telemedicine/ or exp mobile phone/ or exp internet/
6. 4 or 5
7. ((mood or affective or anxiety or panic or eating or "obsessive compulsive" or "attention deficit hyperactivity" or stress or adjustment or personality) adj disorder*).ti,ab.
8. (depress* or dysthymi* or "MDD" or bipolar or "social phobia*" or ADHD or autism or ASD or insomnia or "anorexia nervosa" or "bulimia nervosa" or OCD or schizophrenia or psychosis or PTSD or BPD or EUPD or "self harm" or "self-harm" or suicid*).ti,ab.
9. (mental* adj (health or ill* or disorder*)).ti,ab.
10. ((alcohol* or drug* or substance*) adj (abuse or addict*)).ti,ab.
11. exp mental health/ or exp mental disease/
12. 7 or 8 or 9 or 10 or 11
13. 3 and 6 and 12
14. limit 13 to yr="2005 -Current"

***Cochrane Library*** *(completed October 12^th^, 2021)*

1. ((assess* or diagnostic* or "mood diar*" or PHQ or "PHQ-9" or GAD or "GAD-7" or questionnaire* or screen* or tool* or test* or "computeri?ed adaptive test for mental health" or "CAT-MH" or "e-PASS or WSQ or TAPS or Nview or ada or doctorlink or clinicom) NEAR/5 (accura* or sensitiv* or specific* or "receiver operating characteristic*" or ROC or "area under the curve" or AUC or AUROC or "positive predictive value" or PPV or "negative predictive value" or NPV or precision or recall or "true positive rate" or TPR or "true negative rate" or TNR or valid* or “agreement rate”))
2. mesh descriptor: [Surveys and Questionnaires] explode all trees
3. mesh descriptor: [Sensitivity and Specificity] explode all trees
4. #2 and #3
5. #1 or #4
6. (app or apps or application* or chatbot* or computer* or "conversational agent*" or device* or digital or "e-health" or ehealth or "e-mental health" or "emental health" or electronic or internet or mhealth or "m-health" or mobile* or online or phone* or smartphone* or “smart-phone*” or cellphone* or “cell-phone*” or telehealth or telemedicine or "text messag*" or web or software or algorithm* or tablet* or PC or PCs)
7. MeSH descriptor: [Mobile Applications] this term only
8. MeSH descriptor: [Computers] explode all trees
9. MeSH descriptor: [Telemedicine] this term only
10. MeSH descriptor: [Cell Phone] explode all trees
11. MeSH descriptor: [Internet] explode all trees
12. #6 or #7 or #8 or #9 or #10 or #11
13. ((mood or affective or anxiety or panic or eating or "obsessive compulsive" or "attention deficit hyperactivity" or stress or adjustment or personality) NEXT disorder*)
14. (depress* or dysthymi* or "MDD" or bipolar or "social phobia*" or ADHD or autism or ASD or insomnia or "anorexia nervosa" or "bulimia nervosa" or OCD or schizophrenia or psychosis or PTSD or BPD or EUPD or "self harm" or "self-harm" or suicid*)
15. (mental* NEXT (health or ill* or disorder*))
16. ((alcohol* or drug* or substance*) NEXT (abuse or addict*))
17. MeSH descriptor: [Mental Health] this term only
18. MeSH descriptor: [Mental Disorders] explode all trees
19. #13 or #14 or #15 or #16 or #17 or #18
20. #5 and #12 and #19
21. limit 13 to yr="2005 -Current"

***ASSIA via ProQuest*** *(completed October 12^th^, 2021)*

1. ti(((assess* or diagnostic* or "mood diar*" or PHQ or "PHQ-9" or GAD or "GAD-7" or questionnaire* or screen* or tool* or test* or "computeri?ed adaptive test for mental health" or "CAT-MH" or "e-PASS” or WSQ or TAPS or Nview or ada or doctorlink or clinicom) NEAR/5 (accura* or sensitiv* or specific* or "receiver operating characteristic*" or ROC or "area under the curve" or AUC or AUROC or "positive predictive value" or PPV or "negative predictive value" or NPV or precision or recall or "true positive rate" or TPR or "true negative rate" or TNR or valid* or "agreement rate"))) OR ab(((assess* or diagnostic* or "mood diar*" or PHQ or "PHQ-9" or GAD or "GAD-7" or questionnaire* or screen* or tool* or test* or "computeri?ed adaptive test for mental health" or "CAT-MH" or "e-PASS” or WSQ or TAPS or Nview or ada or doctorlink or clinicom) NEAR/5 (accura* or sensitiv* or specific* or "receiver operating characteristic*" or ROC or "area under the curve" or AUC or AUROC or "positive predictive value" or PPV or "negative predictive value" or NPV or precision or recall or "true positive rate" or TPR or "true negative rate" or TNR or valid* or "agreement rate")))
2. (MAINSUBJECT.EXACT("Questionnaires") OR MAINSUBJECT.EXACT("Psychological tests") OR MAINSUBJECT.EXACT("Psychiatric tests")) AND MAINSUBJECT.EXACT("Receiver operating characteristic analysis")
3. 1 or 2
4. ti((app or apps or application* or chatbot* or computer* or "conversational agent*" or device* or digital or "e-health" or ehealth or "e-mental health" or "emental health" or electronic or internet or mhealth or "m-health" or mobile* or online or phone* or smartphone* or “smart-phone*” or cellphone* or “cell-phone*” or telehealth or telemedicine or "text messag*" or web or software or algorithm* or tablet* or PC or PCs)) OR ab((app or apps or application* or chatbot* or computer* or "conversational agent*" or device* or digital or "e-health" or ehealth or "e-mental health" or "emental health" or electronic or internet or mhealth or "m-health" or mobile* or online or phone* or smartphone* or “smart-phone*” or cellphone* or “cell-phone*” or telehealth or telemedicine or "text messag*" or web or software or algorithm*or tablet* or PC or PCs))
5. MAINSUBJECT.EXACT("Laptop computers") OR MAINSUBJECT.EXACT("Handheld computers") OR MAINSUBJECT.EXACT.EXPLODE("Notebook computers") OR MAINSUBJECT.EXACT("Computers") OR MAINSUBJECT.EXACT.EXPLODE("Telemedicine") OR MAINSUBJECT.EXACT("Mobile phones") OR MAINSUBJECT.EXACT("Internet")
6. 4 or 5
7. ti(((mood or affective or anxiety or panic or eating or "obsessive compulsive" or "attention deficit/hyperactivity" or stress or adjustment or personality) NEAR/1 disorder*)) OR ab(((mood or affective or anxiety or panic or eating or "obsessive compulsive" or "attention deficit/hyperactivity" or stress or adjustment or personality) NEAR/1 disorder*))
8. ti((Depress* or dysthymi* or "MDD" or bipolar or "social phobia*" or ADHD or autism or ASD or insomnia or "anorexia nervosa" or "bulimia nervosa" or OCD or schizophrenia or psychosis or PTSD or BPD or EUPD or "self harm" or "self-harm" or suicid*)) OR ab((Depress* or dysthymi* or "MDD" or bipolar or "social phobia*" or ADHD or autism or ASD or insomnia or "anorexia nervosa" or "bulimia nervosa" or OCD or schizophrenia or psychosis or PTSD or BPD or EUPD or "self harm" or "self-harm" or suicid*))
9. ti((mental* NEAR/1 (health or ill* or disorder*))) OR ab((mental* NEAR/1 (health or ill* or disorder*)))
10. ti(((alcohol* or drug* or substance*) NEAR/1 (abuse or addict*))) OR ab(((alcohol* or drug* or substance*) NEAR/1 (abuse or addict*)))
11. MAINSUBJECT.EXACT("Mental health") OR MAINSUBJECT.EXACT.EXPLODE("Psychiatric disorders")
12. 7 or 8 or 9 or 10 or 11
13. 3 and 6 and 12
14. limit 13 to yr="2005 -Current"

***Web of Science Core Collection*** *(completed October 12^th^, 2021)*

1. ((Assess* or diagnostic* or "mood diar*" or PHQ or "PHQ-9" or GAD or "GAD-7" or questionnaire* or screen* or tool* or test* or "computeri?ed adaptive test for mental health" or "CAT-MH" or "e-PASS” or WSQ or TAPS or Nview or ada or doctorlink or clinicom) NEAR/5 (accura* or sensitiv* or specific* or "receiver operating characteristic*" or ROC or "area under the curve" or AUC or AUROC or "positive predictive value" or PPV or "negative predictive value" or NPV or precision or recall or "true positive rate" or TPR or "true negative rate" or TNR or valid* or “agreement rate”))
2. (App or apps or application* or chatbot* or computer* or "conversational agent*" or device* or digital or "e-health" or ehealth or "e-mental health" or "emental health" or electronic or internet or mhealth or "m-health" or mobile* or online or phone* or smartphone* or “smart-phone*” or cellphone* or “cell-phone*” or telehealth or telemedicine or "text messag*" or web or software or algorithm* or tablet* or PC or PCs)
3. ((mood or affective or anxiety or panic or eating or "obsessive compulsive" or "attention deficit/hyperactivity" or stress or adjustment or personality) NEAR/0 disorder*)
4. (depress* or dysthymi* or "MDD" or bipolar or "social phobia*" or ADHD or autism or ASD or insomnia or "anorexia nervosa" or "bulimia nervosa" or OCD or schizophrenia or psychosis or PTSD or BPD or EUPD or "self harm" or "self-harm" or suicid*)
5. (mental* NEAR/0 (health or ill* or disorder*))
6. ((alcohol* or drug* or substance*) NEAR/0 (abuse or addict*))
7. 3 or 4 or 5 or 6
8. #1 and #2 and #7

**Refined by:** **PUBLICATION YEARS:** (2021 OR 2020 OR 2011 OR 2019 OR 2010 OR 2018 OR 2009 OR 2017 OR 2008 OR 2016 OR 2007 OR 2015 OR 2006 OR 2014 OR 2005 OR 2013 OR 2012 )

***CINAHL via EBSCO*** *(completed October 12^th^, 2021)*

1. TI ( ((assess* or diagnostic* or "mood diar*" or PHQ or "PHQ-9" or GAD or "GAD-7" or questionnaire* or screen* or tool* or test* or "computeri?ed adaptive test for mental health" or "CAT-MH" or "e-PASS" or WSQ or TAPS or Nview or ada or doctorlink or clinicom) N5 (accura* or sensitiv* or specific* or "receiver operating characteristic*" or ROC or "area under the curve" or AUC or AUROC or "positive predictive value" or PPV or "negative predictive value" or NPV or precision or recall or "true positive rate" or TPR or "true negative rate" or TNR or valid* or “agreement rate”)) ) OR AB ( ((assess* or diagnostic* or "mood diar*" or PHQ or "PHQ-9" or GAD or "GAD-7" or questionnaire* or screen* or tool* or test* or "computeri?ed adaptive test for mental health" or "CAT-MH" or "e-PASS” or WSQ or TAPS or Nview or ada or doctorlink or clinicom) N5 (accura* or sensitiv* or specific* or "receiver operating characteristic*" or ROC or "area under the curve" or AUC or AUROC or "positive predictive value" or PPV or "negative predictive value" or NPV or precision or recall or "true positive rate" or TPR or "true negative rate" or TNR or valid* or “agreement rate”)) )
2. ((MH "Surveys+") OR (MH "Questionnaires+")) AND (MH "Sensitivity and Specificity") OR (MH "ROC Curve")
3. 1 or 2
4. TI ( (app or apps or application* or chatbot* or computer* or "conversational agent*" or device* or digital or "e-health" or ehealth or "e-mental health" or "emental health" or electronic or internet or mhealth or "m-health" or mobile* or online or phone* or smartphone* or “smart-phone*” or cellphone* or “cell-phone*” or telehealth or telemedicine or "text messag*" or web or software or algorithm*) ) OR AB ( (app or apps or application* or chatbot* or computer* or "conversational agent*" or device* or digital or "e-health" or ehealth or "e-mental health" or "emental health" or electronic or internet or mhealth or "m-health" or mobile* or online or phone* or smartphone* or “smart-phone*” cellphone* or “cell-phone*”or telehealth or telemedicine or "text messag*" or web or software or algorithm*or tablet* or PC or PCs)
5. (MH "Mobile Applications") OR (MH "Computers and Computerization+") OR (MH "Telemedicine") OR (MH "Cellular Phone+")
6. 4 or 5
7. TI ( ((mood or affective or anxiety or panic or eating or "obsessive compulsive" or "attention deficit hyperactivity" or stress or adjustment or personality) N1 disorder*) ) OR AB ( ((mood or affective or anxiety or panic or eating or "obsessive compulsive" or "attention deficit hyperactivity" or stress or adjustment or personality) N1 disorder*) )
8. TI ( (depress* or dysthymi* or "MDD" or bipolar or "social phobia*" or ADHD or autism or ASD or insomnia or "anorexia nervosa" or "bulimia nervosa" or OCD or schizophrenia or psychosis or PTSD or BPD or EUPD or "self harm" or "self-harm" or suicid*) ) OR AB ( (depress* or dysthymi* or "MDD" or bipolar or "social phobia*" or ADHD or autism or ASD or insomnia or "anorexia nervosa" or "bulimia nervosa" or OCD or schizophrenia or psychosis or PTSD or BPD or EUPD or "self harm" or "self-harm" or suicid*) )
9. TI ( (mental* N1 (health or ill* or disorder*)) ) OR AB ( (mental* N1 (health or ill* or disorder*)) )
10. TI ( ((alcohol* or drug* or substance*) N1 (abuse or addict*)) ) OR AB ( ((alcohol* or drug* or substance*) N1 (abuse or addict*)) )
11. (MH "Mental Health") OR (MH "Mental Disorders+")
12. 7 or 8 or 9 or 10 or 11
13. 3 and 6 and 12
14. limit 13 to yr="2005 -Current"

***PsycINFO via EBSCO*** *(completed October 12^th^, 2021)*

1. TI ( ((Assess* or diagnostic* or "mood diar*" or PHQ or "PHQ-9" or GAD or "GAD-7" or questionnaire* or screen* or tool* or test* or "computeri?ed adaptive test for mental health" or "CAT-MH" or "e-PASS” or WSQ or TAPS or Nview or ada or doctorlink or clinicom) N5 (accura* or sensitiv* or specific* or "receiver operating characteristic*" or ROC or "area under the curve" or AUC or AUROC or "positive predictive value" or PPV or "negative predictive value" or NPV or precision or recall or "true positive rate" or TPR or "true negative rate" or TNR or valid* or “agreement rate”)) ) OR AB ( ((Assess* or diagnostic* or "mood diar*" or PHQ or "PHQ-9" or GAD or "GAD-7" or questionnaire* or screen* or tool* or test* or "computeri?ed adaptive test for mental health" or "CAT-MH" or "e-PASS” or WSQ or TAPS or Nview or ada or doctorlink or clinicom) N5 (accura* or sensitiv* or specific* or "receiver operating characteristic*" or ROC or "area under the curve" or AUC or AUROC or "positive predictive value" or PPV or "negative predictive value" or NPV or precision or recall or "true positive rate" or TPR or "true negative rate" or TNR or valid* or “agreement rate”)) )
2. (DE "Surveys" OR DE "Online Surveys" OR DE "Questionnaires") AND (DE "Test Specificity" OR DE "Test Sensitivity")
3. 1 or 2
4. TI ( (App or apps or application* or chatbot* or computer* or "conversational agent*" or device* or digital or "e-health" or ehealth or "e-mental health" or "emental health" or electronic or internet or mhealth or "m-health" or mobile* or online or phone* or smartphone8 or “smart-phone*” or cellphone* or “cell-phone*” or telehealth or telemedicine or "text messag*" or web or software or algorithm* or tablet* or PC or PCs) ) OR AB ( (App or apps or application* or chatbot* or computer* or "conversational agent*" or device* or digital or "e-health" or ehealth or "e-mental health" or "emental health" or electronic or internet or mhealth or "m-health" or mobile* or online or phone* or smartphone* or “smart-phone*” or cellphone* or “cell-phone*” or telehealth or telemedicine or "text messag*" or web or software or algorithm* or tablet* or PC or PCs) )
5. DE "Mobile Applications" OR DE "Computer Applications" OR DE "Mobile Phones" OR DE "Smartphones" OR DE "Computers" OR DE "Tablet Computers" OR DE "Telemedicine" OR DE "Mobile Health"
6. 4 or 5
7. TI ( ((mood or affective or anxiety or panic or eating or "obsessive compulsive" or "attention deficit/hyperactivity" or stress or adjustment or personality) N1 disorder*) ) OR AB ( ((mood or affective or anxiety or panic or eating or "obsessive compulsive" or "attention deficit/hyperactivity" or stress or adjustment or personality) N1 disorder*) )
8. TI ( (Depress* or dysthymi* or "MDD" or bipolar or "social phobia*" or ADHD or autism or ASD or insomnia or "anorexia nervosa" or "bulimia nervosa" or OCD or schizophrenia or psychosis or PTSD or BPD or EUPD or "self harm" or "self-harm" or suicid*) ) OR AB ( (Depress* or dysthymi* or "MDD" or bipolar or "social phobia*" or ADHD or autism or ASD or insomnia or "anorexia nervosa" or "bulimia nervosa" or OCD or schizophrenia or psychosis or PTSD or BPD or EUPD or "self harm" or "self-harm" or suicid*) )
9. TI ( (mental* N1 (health or ill* or disorder*)) ) OR AB ( (mental* N1 (health or ill* or disorder*)) )
10. TI ( ((alcohol* or drug* or substance*) N1 (abuse or addict*)) ) OR AB ( ((alcohol* or drug* or substance*) N1 (abuse or addict*)) )
11. DE "Mental Health" OR DE "Mental Disorders" OR DE "Affective Disorders" OR DE "Anxiety Disorders" OR DE "Autism Spectrum Disorders" OR DE "Bipolar Disorder" OR DE "Borderline States" DE "Eating Disorders" OR DE "Neurodevelopmental Disorders" OR DE "Personality Disorders" OR DE "Psychosis" OR DE "Serious Mental Illness" OR DE "Sleep Wake Disorders" OR DE "Stress and Trauma Related Disorders" OR DE "Substance Related and Addictive Disorders"
12. 7 or 8 or 9 or 10 or 11
13. 3 and 6 and 12
14. limit 13 to yr="2005 -Current"

**Grey literature:**

***ClinicalTrials.gov*** *(completed October 12^th^, 2021)*
Condition: “mental health”; Other terms: “assessment tools”

***NIHR UK trials*** *(completed October 12^th^, 2021)*

Search term: “mental health assessment”

***Advanced Google search*** *(completed October 12^th^, 2021)*

Search terms: "digital tools" AND "mental health" AND “assessment” AND “questionnaire” (site:.edu OR site:.ac OR site:.gov OR site:.org)
Only the first 50 pages were searched.

***Hand searching relevant systematic reviews and meta-analyses*** *(completed October 21^st^, 2021)*
