# Supplementary material for: The Current State and Validity of Digital Assessment Tools for Psychiatry: Systematic Review
Source: JMIR Ment Health. 2022 Mar 30;9(3):e32824. doi: 10.2196/32824 (PMC9008525; doi:10.2196/32824)
Supplement: Multimedia Appendix 2 [file mental_v9i3e32824_app2.docx]

***Multimedia Appendix 2***

**Supplementary Table 1.** Checklist summary of mental health disorders investigated in the included studies.

| Disorder  Study | Any mood/anxiety disorder | Any mood disorder | Any anxiety disorder | Any depressive disorder | GAD | Panic disorder | Social phobia | PTSD | OCD | Agora-phobia | MDD | BD/Bipolar spectrum disorder | ADHD | AUD/SUD | Eating disorders^1^ | EUPD | Psychosis | Suicidality |
| --- | --- | --- | --- | --- | --- | --- | --- | --- | --- | --- | --- | --- | --- | --- | --- | --- | --- | --- |
| Achtyes et al., 2015^α^ |  |  |  |  |  |  |  |  |  |  | 🗸 |  |  |  |  |  |  |  |
| Ballester et al., 2019 |  | 🗸 | 🗸 | 🗸 | 🗸 | 🗸 |  |  |  |  |  |  |  |  |  |  |  |  |
| Cano-Vindel et al., 2018 |  |  |  |  | 🗸 |  |  |  |  |  | 🗸 |  |  |  |  |  |  |  |
| Donker et al., 2009 |  |  |  | 🗸 | 🗸 | 🗸 | 🗸 | 🗸 | 🗸 | 🗸 |  |  |  | 🗸  (AUD) |  |  |  |  |
| Donker et al., 2010 |  |  |  | 🗸 |  |  |  |  |  |  |  |  |  |  |  |  |  |  |
| Donker et al., 2011 |  |  | 🗸 | 🗸 | 🗸 | 🗸 | 🗸 | 🗸 |  |  |  |  |  |  |  |  |  |  |
| Du et al., 2017 |  |  |  |  |  |  |  |  |  |  | 🗸 |  |  |  |  |  |  |  |
| Fowler et al., 2018 |  |  |  |  |  |  |  |  |  |  |  |  |  |  |  | 🗸 |  |  |
| Gaynes et al., 2010 | 🗸 |  | 🗸 | 🗸 |  |  |  | 🗸 |  |  |  | 🗸  (bipolar spectrum disorder) |  |  |  |  |  |  |
| Gibbons et al., 2012 |  |  |  | 🗸 |  |  |  |  |  |  | 🗸 |  |  |  |  |  |  |  |
| Gibbons et al., 2013 |  |  |  |  |  |  |  |  |  |  | 🗸 |  |  |  |  |  |  |  |
| Gibbons et al., 2014 |  |  |  |  | 🗸 |  |  |  |  |  | 🗸 |  |  |  |  |  |  |  |
| Graham et al., 2019 |  |  |  |  | 🗸 |  |  |  |  |  | 🗸 |  |  |  |  |  |  |  |
| Guinart et al., 2020 |  |  |  |  |  |  |  |  |  |  |  |  |  |  |  |  | 🗸 |  |
| Kertz et al., 2013 |  |  |  |  | 🗸 |  |  |  |  |  |  |  |  |  |  |  |  |  |
| Kim et al., 2021 |  |  |  |  | 🗸 |  |  |  |  |  |  |  |  |  |  |  |  |  |
| Lohanan et al., 2020 |  |  |  |  |  |  |  |  |  |  |  |  |  |  |  | 🗸 |  |  |
| McNeely et al., 2015 |  |  |  |  |  |  |  |  |  |  |  |  |  | 🗸  (All) |  |  |  |  |
| Meuldijk et al., 2017 |  |  |  | 🗸 | 🗸 | 🗸 | 🗸 | 🗸 | 🗸 | 🗸 |  |  |  | 🗸  (AUD) |  |  |  |  |
| Munoz-Navarro et al., 2017 |  |  |  |  | 🗸 |  |  |  |  |  |  |  |  |  |  |  |  |  |
| Nguyen et al., 2015 |  |  |  |  | 🗸 | 🗸 | 🗸 | 🗸 | 🗸 |  | 🗸 |  |  | 🗸 (AUD) | 🗸  (BN) |  |  |  |
| Nielsen et al., 2017 |  |  |  |  |  |  |  |  |  |  | 🗸 |  |  |  |  |  |  |  |
| Oromendia et al., 2015 |  |  |  |  |  | 🗸 |  |  |  |  |  |  |  |  |  |  |  |  |
| Rogers et al., 2021 |  |  |  | 🗸 | 🗸 | 🗸 | 🗸 |  |  |  |  | 🗸  (BD) |  | 🗸 (SUD) |  |  |  | 🗸 |
| Sanchez et al., 2021 |  |  |  |  |  |  |  |  |  |  |  |  |  | 🗸  (AUD) |  |  |  |  |
| Schulte-van Maaren et al., 2013 |  |  | 🗸 |  |  |  |  |  |  |  |  |  |  |  |  |  |  |  |
| ter Huurne et al., 2015 |  |  |  |  |  |  |  |  |  |  |  |  |  |  | 🗸  (All) |  |  |  |
| Yoon et al., 2020 |  |  |  |  |  |  |  |  |  |  |  |  |  |  |  |  |  | 🗸 |

***Note.*** ^1^ Comprises AN, BN, BED, and EDNOS; *^α^* The authors also looked at GAD and BD but no diagnostic accuracy data was reported.
***Key.*** ADHD, attention deficit hyperactivity disorder; AN, anorexia nervosa; AUD, alcohol use disorder; BD, bipolar disorder; BED, binge eating disorder; EDNOS, eating disorder not otherwise specified; EUPD, emotionally unstable personality disorder; GAD, generalized anxiety disorder; MDD, major depressive disorder; OCD, obsessive compulsive disorder; PTSD, post-traumatic stress disorder; SUD, substance use disorder.
