# Supplementary material for: The Current State and Validity of Digital Assessment Tools for Psychiatry: Systematic Review
Source: JMIR Ment Health. 2022 Mar 30;9(3):e32824. doi: 10.2196/32824 (PMC9008525; doi:10.2196/32824)
Supplement: Multimedia Appendix 3 [file mental_v9i3e32824_app3.docx]

***Multimedia Appendix 3***

**Supplementary Table 2.** Diagnostic accuracy per index test separated by condition of interest.

| **Study** | | **Index test** | **Cut-off** | **Sensitivity** | **Sensitivity 95% CI** | **Specificity** |  | **Specificity 95% CI** | **Youden’s index** | **AUC** | **AUC 95% CI** |
| --- | --- | --- | --- | --- | --- | --- | --- | --- | --- | --- | --- |
|  | **Any mood or anxiety disorder** | | | | | | | | | | |
| Gaynes et al., 2010 | | M-3 | 5 | 0.83 | 0.77-0.88 | 0.76 |  | 0.72-0.80 | 0.59 | - | - |
|  | **Any mood disorder** | | | | | | | | | | |
| Ballester et al., 2019 | | WMH-ICS survey (12 months) | - | 0.76 | - | 0.80 |  | - | 0.56 | 0.78 | - |
|  |  | WMH-ICS survey (lifetime) | - | 0.95 | - | 0.60 |  | - | 0.60 | 0.77 | - |
|  | **Any anxiety disorder** | | | | | | | | | | |
| Ballester et al., 2019 | | WMH-ICS survey (12 months) | - | 0.79 | - | 0.89 |  | - | 0.68 | 0.84 | - |
|  |  | WMH-ICS survey (lifetime) | - | 0.92 | - | 0.71 |  | - | 0.63 | 0.81 | - |
| Donker et al., 2011 | | GAD-7 | 5 | 0.89 | 0.75-0.96 | 0.17 |  | 0.12-0.25 | 0.06 | 0.77 | 0.68-0.85 |
|  |  |  | 7 | 0.75 | 0.59-0.86 | 0.32 |  | 0.25-0.41 | 0.07 |  |  |
|  |  |  | 9 | 0.72 | 0.56-0.48 | 0.38 |  | 0.30-0.47 | 0.10 |  |  |
|  |  |  | 10 | 0.64 | 0.48-0.78 | 0.46 |  | 0.37-0.54 | 0.10 |  |  |
|  |  |  | 11 | 0.58 | 0.42-0.73 | 0.51 |  | 0.42-0.60 | 0.09 |  |  |
|  |  |  | 12 | 0.50 | 0.34-0.66 | 0.57 |  | 0.48-0.66 | 0.07 |  |  |
|  |  |  | 13 | 0.42 | 0.27-0.58 | 0.63 |  | 0.54-0.71 | 0.05 |  |  |
|  |  |  | 14 | 0.42 | 0.27-0.58 | 0.71 |  | 0.62-0.78 | 0.13 |  |  |
|  |  |  | 15 | 0.36 | 0.22-0.52 | 0.78 |  | 0.69-0.84 | 0.14 |  |  |
|  |  |  | 16 | 0.25 | 0.14-0.41 | 0.83 |  | 0.76-0.89 | 0.08 |  |  |
|  |  |  | 17 | 0.17 | 0.07-0.32 | 0.85 |  | 0.78-0.90 | 0.02 |  |  |
|  |  | GAD-2 | 2 | 0.86 | 0.71-0.94 | 0.22 |  | 0.15-0.30 | 0.08 | 0.76 | 0.68-0.84 |
|  |  |  | 3 | 0.72 | 0.56-0.84 | 0.39 |  | 0.31-0.48 | 0.11 |  |  |
|  |  |  | 4 | 0.58 | 0.42-0.73 | 0.56 |  | 0.47-0.65 | 0.14 |  |  |
|  |  |  | 5 | 0.47 | 0.32-0.63 | 0.72 |  | 0.63-0.79 | 0.19 |  |  |
|  |  |  | 6 | 0.22 | 0.12-0.38 | 0.83 |  | 0.75-0.88 | 0.05 |  |  |
|  |  | GAD-SI | 2 | 0.72 | 0.56-0.84 | 0.41 |  | 0.32-0.49 | 0.13 | 0.78 | 0.69-0.86 |
|  |  |  | 3 | 0.36 | 0.22-0.52 | 0.68 |  | 0.59-0.75 | 0.04 |  |  |
| Gaynes et al., 2010 | | M-3 | 3 | 0.82 | 0.75-0.87 | 0.78 |  | 0.74-0.81 | 0.60 | - | - |
| Schulte-van Maaren et al., 2013 | | BSA | 8.5 | 0.88 | **-** | 0.88 |  | **-** | 0.76 | 0.95 | **-** |
|  |  | PI-R | 30.5 | 0.86 | **-** | 0.86 |  | **-** | 0.72 | 0.94 | **-** |
|  |  | PAI | 21.5 | 0.86 | **-** | 0.86 |  | **-** | 0.72 | 0.93 | **-** |
|  |  | PSWQ | 55.5 | 0.87 | **-** | 0.87 |  | **-** | 0.74 | 0.93 | **-** |
|  |  | WDQ | 55.5 | 0.86 | **-** | 0.85 |  | **-** | 0.71 | 0.92 | **-** |
|  |  | SIAS | 24.5 | 0.89 | **-** | 0.89 |  | **-** | 0.78 | 0.96 | **-** |
|  |  | SPS | 14 | 0.90 | **-** | 0.90 |  | **-** | 0.80 | 0.96 | **-** |
|  |  | IES-R | 27.5 | 0.91 | **-** | 0.91 |  | **-** | 0.82 | 0.96 | **-** |
|  | **Any depressive disorder** | | | | | | | | | | |
| Ballester et al., 2019 | | WMH-ICS survey (12 months) | 1 | 0.98 | **-** | 0.65 |  | **-** | 0.63 | 0.81 | **-** |
|  |  |  | 2 | 0.98 | **-** | 0.65 |  | **-** | 0.63 | 0.81 | **-** |
|  |  |  | 3 | 0.98 | **-** | 0.65 |  | **-** | 0.63 | 0.81 | **-** |
|  |  |  | 4 | 0.98 | **-** | 0.65 |  | **-** | 0.63 | 0.81 | **-** |
|  |  |  | 5 | 0.98 | **-** | 0.65 |  | **-** | 0.63 | 0.81 | **-** |
|  |  |  | 6 | 0.98 | **-** | 0.65 |  | **-** | 0.63 | 0.81 | **-** |
|  |  |  | 7 | 0.98 | **-** | 0.65 |  | **-** | 0.63 | 0.81 | **-** |
|  |  |  | 8 | 0.98 | **-** | 0.66 |  | **-** | 0.64 | 0.82 | **-** |
|  |  |  | 9 | 0.98 | **-** | 0.67 |  | **-** | 0.65 | 0.83 | **-** |
|  |  |  | 10 | 0.98 | **-** | 0.67 |  | **-** | 0.65 | 0.83 | **-** |
|  |  |  | 11 | 0.93 | **-** | 0.70 |  | **-** | 0.63 | 0.81 | **-** |
|  |  |  | 12 | 0.93 | **-** | 0.72 |  | **-** | 0.65 | 0.82 | **-** |
|  |  |  | 13 | 0.93 | **-** | 0.83 |  | **-** | 0.76 | 0.88 | **-** |
|  |  |  | 14 | 0.73 | **-** | 0.87 |  | **-** | 0.60 | 0.80 | **-** |
|  |  |  | 15 | 0.71 | **-** | 0.90 |  | **-** | 0.61 | 0.80 | **-** |
|  |  |  | 16 | 0.60 | **-** | 0.92 |  | **-** | 0.52 | 0.76 | **-** |
|  |  |  | 17 | 0.50 | **-** | 0.92 |  | **-** | 0.42 | 0.71 | **-** |
|  |  |  | 18 | 0.47 | **-** | 0.94 |  | **-** | 0.41 | 0.70 | **-** |
|  |  |  | 19 | 0.44 | **-** | 0.97 |  | **-** | 0.41 | 0.70 | **-** |
|  |  |  | 20 | 0.26 | **-** | 0.97 |  | **-** | 0.23 | 0.62 | **-** |
|  |  |  | 21 | 0.16 | **-** | 0.97 |  | **-** | 0.13 | 0.57 | **-** |
|  |  |  | 22 | 0.06 | **-** | 0.98 |  | **-** | 0.04 | 0.52 | **-** |
|  |  |  | 23 | 0.06 | **-** | 0.99 |  | **-** | 0.05 | 0.52 | **-** |
|  |  |  | 24 | 0.03 | **-** | 0.99 |  | **-** | 0.02 | 0.51 | **-** |
|  |  | WMH-ICS survey (lifetime) | 1 | 0.99 | **-** | 0.46 |  | **-** | 0.45 | 0.73 | **-** |
|  |  |  | 2 | 0.99 | **-** | 0.46 |  | **-** | 0.45 | 0.73 | **-** |
|  |  |  | 3 | 0.99 | **-** | 0.46 |  | **-** | 0.45 | 0.73 | **-** |
|  |  |  | 4 | 0.99 | **-** | 0.46 |  | **-** | 0.45 | 0.73 | **-** |
|  |  |  | 5 | 0.99 | **-** | 0.46 |  | **-** | 0.45 | 0.73 | **-** |
|  |  |  | 6 | 0.99 | **-** | 0.46 |  | **-** | 0.45 | 0.73 | **-** |
|  |  |  | 7 | 0.99 | **-** | 0.46 |  | **-** | 0.45 | 0.73 | **-** |
|  |  |  | 8 | 0.99 | **-** | 0.48 |  | **-** | 0.47 | 0.73 | **-** |
|  |  |  | 9 | 0.99 | **-** | 0.49 |  | **-** | 0.48 | 0.74 | **-** |
|  |  |  | 10 | 0.97 | **-** | 0.50 |  | **-** | 0.47 | 0.74 | **-** |
|  |  |  | 11 | 0.97 | **-** | 0.54 |  | **-** | 0.51 | 0.76 | **-** |
|  |  |  | 12 | 0.96 | **-** | 0.58 |  | **-** | 0.54 | 0.77 | **-** |
|  |  |  | 13 | 0.96 | **-** | 0.65 |  | **-** | 0.61 | 0.80 | **-** |
|  |  |  | 14 | 0.89 | **-** | 0.70 |  | **-** | 0.59 | 0.79 | **-** |
|  |  |  | 15 | 0.62 | **-** | 0.73 |  | **-** | 0.35 | 0.68 | **-** |
|  |  |  | 16 | 0.54 | **-** | 0.78 |  | **-** | 0.32 | 0.66 | **-** |
|  |  |  | 17 | 0.49 | **-** | 0.84 |  | **-** | 0.33 | 0.66 | **-** |
|  |  |  | 18 | 0.47 | **-** | 0.87 |  | **-** | 0.34 | 0.67 | **-** |
|  |  |  | 19 | 0.44 | **-** | 0.92 |  | **-** | 0.36 | 0.68 | **-** |
|  |  |  | 21 | 0.34 | **-** | 0.93 |  | **-** | 0.27 | 0.63 | **-** |
|  |  |  | 21 | 0.25 | **-** | 0.94 |  | **-** | 0.19 | 0.60 | **-** |
|  |  |  | 22 | 0.16 | **-** | 0.95 |  | **-** | 0.11 | 0.55 | **-** |
|  |  |  | 23 | 0.12 | **-** | 0.96 |  | **-** | 0.08 | 0.54 | **-** |
|  |  |  | 24 | 0.07 | **-** | 0.99 |  | **-** | 0.06 | 0.53 | **-** |
| Donker et al., 2009 | | WSQ | Q1≥5 and Q2=1 | 0.85 | **-** | 0.59 |  | **-** | 0.44 | 0.72 | 0.64-0.80 |
|  |  | CES-D | - | **-** | **-** | **-** |  | **-** | **-** | 0.84 | 0.77-0.90 |
| Donker et al., 2010 | | SID | 1 | 1.00 | **-** | 0.07 |  | **-** | 0.07 | 0.71 | 0.63-0.79 |
|  |  |  | 2 | 1.00 | **-** | 0.11 |  | **-** | 0.11 |  |  |
|  |  |  | 3 | 1.00 | **-** | 0.22 |  | **-** | 0.22 |  |  |
|  |  |  | 4 | 0.96 | **-** | 0.32 |  | **-** | 0.28 |  |  |
|  |  |  | 5 | 0.87 | **-** | 0.51 |  | **-** | 0.38 |  |  |
|  |  |  | 6 | 0.48 | **-** | 0.72 |  | **-** | 0.20 |  |  |
|  |  |  | 7 | 0.23 | **-** | 0.89 |  | **-** | 0.12 |  |  |
|  |  |  | 8 | 0.08 | **-** | 0.98 |  | **-** | 0.06 |  |  |
|  |  |  | 9 | 0.00 | **-** | 1.00 |  | **-** | 0.00 |  |  |
|  |  | CES-D | 15 | 0.98 | **-** | 0.31 |  | **-** | 0.29 | 0.84 | 0.77-0.90 |
|  |  |  | 16 | 0.98 | **-** | 0.34 |  | **-** | 0.32 |  |  |
|  |  |  | 17 | 0.96 | **-** | 0.43 |  | **-** | 0.39 |  |  |
|  |  |  | 18 | 0.96 | **-** | 0.49 |  | **-** | 0.45 |  |  |
|  |  |  | 19 | 0.96 | **-** | 0.54 |  | **-** | 0.50 |  |  |
|  |  |  | 20 | 0.94 | **-** | 0.56 |  | **-** | 0.50 |  |  |
|  |  |  | 21 | 0.94 | **-** | 0.69 |  | **-** | 0.63 |  |  |
|  |  |  | 22 | 0.94 | **-** | 0.62 |  | **-** | 0.56 |  |  |
|  |  |  | 23 | 0.90 | **-** | 0.62 |  | **-** | 0.52 |  |  |
|  |  |  | 24 | 0.85 | **-** | 0.63 |  | **-** | 0.48 |  |  |
|  |  |  | 25 | 0.83 | **-** | 0.64 |  | **-** | 0.47 |  |  |
|  |  |  | 26 | 0.83 | **-** | 0.68 |  | **-** | 0.51 |  |  |
|  |  |  | 27 | 0.79 | **-** | 0.70 |  | **-** | 0.49 |  |  |
|  |  |  | 28 | 0.73 | **-** | 0.71 |  | **-** | 0.44 |  |  |
|  |  |  | 29 | 0.73 | **-** | 0.72 |  | **-** | 0.45 |  |  |
|  |  |  | 30 | 0.73 | **-** | 0.76 |  | **-** | 0.49 |  |  |
|  |  |  | 31 | 0.72 | **-** | 0.79 |  | **-** | 0.51 |  |  |
|  |  |  | 32 | 0.65 | **-** | 0.82 |  | **-** | 0.47 |  |  |
|  |  | K10 | 18 | 0.98 | **-** | 0.20 |  | **-** | 0.18 | 0.81 | 0.73-0.88 |
|  |  |  | 19 | 0.98 | **-** | 0.24 |  | **-** | 0.22 |  |  |
|  |  |  | 20 | 0.98 | **-** | 0.25 |  | **-** | 0.23 |  |  |
|  |  |  | 21 | 0.98 | **-** | 0.31 |  | **-** | 0.29 |  |  |
|  |  |  | 22 | 0.94 | **-** | 0.34 |  | **-** | 0.28 |  |  |
|  |  |  | 23 | 0.92 | **-** | 0.42 |  | **-** | 0.34 |  |  |
|  |  |  | 24 | 0.87 | **-** | 0.46 |  | **-** | 0.33 |  |  |
|  |  |  | 25 | 0.85 | **-** | 0.49 |  | **-** | 0.34 |  |  |
|  |  |  | 26 | 0.83 | **-** | 0.51 |  | **-** | 0.34 |  |  |
|  |  |  | 27 | 0.81 | **-** | 0.57 |  | **-** | 0.38 |  |  |
|  |  |  | 28 | 0.81 | **-** | 0.60 |  | **-** | 0.41 |  |  |
|  |  |  | 29 | 0.81 | **-** | 0.67 |  | **-** | 0.48 |  |  |
|  |  |  | 30 | 0.75 | **-** | 0.71 |  | **-** | 0.46 |  |  |
|  |  |  | 31 | 0.71 | **-** | 0.77 |  | **-** | 0.48 |  |  |
|  |  |  | 32 | 0.69 | **-** | 0.79 |  | **-** | 0.48 |  |  |
|  |  |  | 33 | 0.62 | **-** | 0.84 |  | **-** | 0.46 |  |  |
|  |  |  | 34 | 0.52 | **-** | 0.86 |  | **-** | 0.38 |  |  |
|  |  |  | 35 | 0.48 | **-** | 0.87 |  | **-** | 0.35 |  |  |
| Donker et al., 2011 | | GAD-7 | 5 | 0.97 | 0.85-0.99 | 0.19 |  | 0.13-0.27 | 0.16 | 0.57 | 0.47-0.68 |
|  |  |  | 7 | 0.94 | 0.80-0.98 | 0.37 |  | 0.29-0.45 | 0.31 |  |  |
|  |  |  | 9 | 0.85 | 0.69-0.93 | 0.41 |  | 0.33-0.50 | 0.26 |  |  |
|  |  |  | 10 | 0.76 | 0.59-0.87 | 0.48 |  | 0.40-0.57 | 0.24 |  |  |
|  |  |  | 11 | 0.76 | 0.59-0.88 | 0.56 |  | 0.47-0.64 | 0.32 |  |  |
|  |  |  | 12 | 0.64 | 0.47-0.78 | 0.60 |  | 0.52-0.69 | 0.24 |  |  |
|  |  |  | 13 | 0.58 | 0.41-0.73 | 0.67 |  | 0.58-0.75 | 0.25 |  |  |
|  |  |  | 14 | 0.55 | 0.38-0.70 | 0.74 |  | 0.66-0.81 | 0.29 |  |  |
|  |  |  | 15 | 0.49 | 0.33-0.65 | 0.81 |  | 0.73-0.87 | 0.30 |  |  |
|  |  |  | 16 | 0.39 | 0.25-0.56 | 0.87 |  | 0.80-0.92 | 0.26 |  |  |
|  |  |  | 17 | 0.30 | 0.17-0.47 | 0.89 |  | 0.82-0.93 | 0.19 |  |  |
|  |  | GAD-2 | 2 | 0.97 | 0.85-0.99 | 0.24 |  | 0.18-0.32 | 0.21 | 0.59 | 0.49-0.70 |
|  |  |  | 3 | 0.88 | 0.73-0.95 | 0.43 |  | 0.34-0.52 | 0.31 |  |  |
|  |  |  | 4 | 0.64 | 0.47-0.78 | 0.57 |  | 0.48-0.66 | 0.21 |  |  |
|  |  |  | 5 | 0.61 | 0.44-0.75 | 0.75 |  | 0.67-0.82 | 0.36 |  |  |
|  |  |  | 6 | 0.36 | 0.22-0.54 | 0.86 |  | 0.79-0.91 | 0.22 |  |  |
|  |  | GAD-SI | 2 | 0.82 | 0.66-0.91 | 0.43 |  | 0.34-0.52 | 0.25 | 0.57 | 0.47-0.67 |
|  |  |  | 3 | 0.49 | 0.33-0.65 | 0.71 |  | 0.62-0.78 | 0.20 |  |  |
| Gaynes et al., 2010 | | M-3 | 5 | 0.84 | 0.77-0.89 | 0.80 |  | 0.76-0.83 | 0.64 | - | - |
| Gibbons et al., 2012 | | CAT-DI | -0.61 (non-psychiatric sample as comparator) | 0.90 | **-** | 0.88 |  | **-** | 0.78 | **-** | **-** |
|  |  |  | -0.61 (psychiatric comparator) | 0.90 | **-** | 0.64 |  | **-** | 0.54 | **-** | **-** |
| Meuldijk et al., 2017 | | WSQ | Q1≥5 and Q2=1 | 0.58 |  | 0.94 |  |  | 0.52 | 0.83 | **-** |
| Rogers et al., 2021 | | CMFC initial screen | - | 0.94 | 0.82-0.98 | 0.65 |  | 0.57-0.72 | 0.59 | - | **-** |
|  |  | CMFC SAM | - | 0.45 | 0.31-0.60 | 0.93 |  | 0.88-0.96 | 0.38 | - | **-** |
|  |  | CMFC SAM (preliminary) | - | 0.73 | 0.54-0.87 | 0.92 |  | 0.87-0.96 | 0.65 | - | **-** |
|  | **Generalized anxiety disorder** | | | | | | | | | | |
| Ballester et al., 2019 | | WMH-ICS survey (12 months) | 13 | 1.00 | **-** | 0.81 |  | **-** | 0.81 | 0.91 | **-** |
|  |  |  | 14 | 1.00 | **-** | 0.81 |  | **-** | 0.81 | 0.91 | **-** |
|  |  |  | 15 | 1.00 | **-** | 0.81 |  | **-** | 0.81 | 0.91 | **-** |
|  |  |  | 16 | 1.00 | **-** | 0.82 |  | **-** | 0.82 | 0.91 | **-** |
|  |  |  | 17 | 1.00 | **-** | 0.83 |  | **-** | 0.83 | 0.91 | **-** |
|  |  |  | 18 | 1.00 | **-** | 0.83 |  | **-** | 0.83 | 0.91 | **-** |
|  |  |  | 19 | 1.00 | **-** | 0.83 |  | **-** | 0.83 | 0.92 | **-** |
|  |  |  | 20 | 1.00 | **-** | 0.85 |  | **-** | 0.85 | 0.92 | **-** |
|  |  |  | 21 | 1.00 | **-** | 0.86 |  | **-** | 0.86 | 0.93 | **-** |
|  |  |  | 22 | 0.85 | **-** | 0.88 |  | **-** | 0.73 | 0.92 | **-** |
|  |  |  | 23 | 0.95 | **-** | 0.89 |  | **-** | 0.84 | 0.92 | **-** |
|  |  |  | 24 | 0.95 | **-** | 0.90 |  | **-** | 0.85 | 0.92 | **-** |
|  |  |  | 25 | 0.73 | **-** | 0.92 |  | **-** | 0.65 | 0.92 | **-** |
|  |  |  | 26 | 0.63 | **-** | 0.93 |  | **-** | 0.56 | 0.82 | **-** |
|  |  |  | 27 | 0.63 | **-** | 0.94 |  | **-** | 0.57 | 0.78 | **-** |
|  |  |  | 28 | 0.63 | **-** | 0.94 |  | **-** | 0.57 | 0.79 | **-** |
|  |  |  | 29 | 0.63 | **-** | 0.96 |  | **-** | 0.59 | 0.80 | **-** |
|  |  |  | 30 | 0.63 | **-** | 0.97 |  | **-** | 0.60 | 0.80 | **-** |
|  |  |  | 31 | 0.41 | **-** | 0.99 |  | **-** | 0.40 | 0.70 | **-** |
|  |  |  | 32 | 0.32 | **-** | 0.99 |  | **-** | 0.31 | 0.65 | **-** |
|  |  | WMH-ICS survey (lifetime) | 13 | 1.00 | - | 0.58 |  | - | 0.58 | 0.79 | **-** |
|  |  |  | 14 | 1.00 | - | 0.58 |  | - | 0.58 | 0.79 | **-** |
|  |  |  | 15 | 1.00 | - | 0.60 |  | - | 0.60 | 0.80 | **-** |
|  |  |  | 16 | 1.00 | - | 0.60 |  | - | 0.60 | 0.80 | **-** |
|  |  |  | 17 | 1.00 | - | 0.61 |  | - | 0.61 | 0.80 | **-** |
|  |  |  | 18 | 1.00 | - | 0.61 |  | - | 0.61 | 0.81 | **-** |
|  |  |  | 19 | 1.00 | - | 0.68 |  | - | 0.68 | 0.84 | **-** |
|  |  |  | 20 | 1.00 | - | 0.70 |  | - | 0.70 | 0.85 | **-** |
|  |  |  | 21 | 1.00 | - | 0.71 |  | - | 0.71 | 0.86 | **-** |
|  |  |  | 22 | 0.97 | - | 0.75 |  | - | 0.72 | 0.86 | **-** |
|  |  |  | 23 | 0.97 | - | 0.77 |  | - | 0.74 | 0.87 | **-** |
|  |  |  | 24 | 0.97 | - | 0.79 |  | - | 0.76 | 0.88 | **-** |
|  |  |  | 25 | 0.85 | - | 0.83 |  | - | 0.68 | 0.84 | **-** |
|  |  |  | 26 | 0.85 | - | 0.86 |  | - | 0.71 | 0.85 | **-** |
|  |  |  | 27 | 0.85 | - | 0.90 |  | - | 0.75 | 0.86 | **-** |
|  |  |  | 28 | 0.64 | - | 0.94 |  | - | 0.58 | 0.77 | **-** |
|  |  |  | 29 | 0.53 | - | 0.96 |  | - | 0.49 | 0.73 | **-** |
|  |  |  | 30 | 0.53 | - | 0.96 |  | - | 0.49 | 0.74 | **-** |
|  |  |  | 31 | 0.53 | - | 0.97 |  | - | 0.50 | 0.75 | **-** |
|  |  |  | 32 | 0.36 | - | 0.98 |  | - | 0.34 | 0.67 | **-** |
| Cano-Vindel et al., 2018 | | GAD-2 | 0 | 1.00 | 0.97-1.00 | 0.00 |  | 0.00-0.09 | 0.00 | - | - |
|  |  |  | 1 | 0.98 | 0.94-0.99 | 0.07 |  | 0.03-0.19 | 0.05 | - | - |
|  |  |  | 2 | 0.96 | 0.92-0.98 | 0.22 |  | 0.12-0.37 | 0.18 | - | - |
|  |  |  | 3 | 0.88 | 0.81-0.92 | 0.61 |  | 0.46-0.74 | 0.49 | - | - |
|  |  |  | 4 | 0.77 | 0.70-0.84 | 0.80 |  | 0.66-0.90 | 0.57 | - | - |
|  |  |  | 5 | 0.56 | 0.48-0.64 | 0.85 |  | 0.72-0.93 | 0.41 | - | - |
|  |  |  | 6 | 0.38 | 0.30-0.46 | 0.90 |  | 0.77-0.96 | 0.28 | - | - |
| Donker et al., 2009 | | WSQ | Q3≥2 | 0.93 | **-** | 0.45 |  | **-** | 0.38 | 0.78 | 0.69-0.86 |
|  |  | GAD-7 | - | **-** | **-** | **-** |  | **-** | **-** | 0.77 | 0.68-0.85 |
| Donker et al., 2011 | | GAD-7 | 5 | 1.00 | 0.89-1.00 | 0.20 |  | 0.14-0.27 | 0.20 | 0.77 | 0.68-0.85 |
|  |  |  | 6 | 0.97 | 0.83-0.99 | 0.28 |  | 0.21-0.36 | 0.25 |  |  |
|  |  |  | 7 | 0.93 | 0.79-0.98 | 0.32 |  | 0.28-0.45 | 0.25 |  |  |
|  |  |  | 8 | 0.93 | 0.79-0.98 | 0.38 |  | 0.30-0.47 | 0.31 |  |  |
|  |  |  | 9 | 0.93 | 0.79-0.98 | 0.43 |  | 0.34-0.51 | 0.36 |  |  |
|  |  |  | 10 | 0.87 | 0.70-0.95 | 0.50 |  | 0.42-0.59 | 0.37 |  |  |
|  |  |  | 11 | 0.83 | 0.70-0.95 | 0.57 |  | 0.48-0.65 | 0.40 |  |  |
|  |  |  | 12 | 0.83 | 0.66-0.93 | 0.65 |  | 0.56-0.72 | 0.48 |  |  |
|  |  |  | 13 | 0.73 | 0.56-0.86 | 0.70 |  | 0.62-0.77 | 0.43 |  |  |
|  |  |  | 14 | 0.63 | 0.45-0.78 | 0.76 |  | 0.67-0.82 | 0.39 |  |  |
|  |  |  | 15 | 0.50 | 0.33-0.67 | 0.80 |  | 0.73-0.86 | 0.30 |  |  |
|  |  |  | 16 | 0.40 | 0.25-0.58 | 0.87 |  | 0.80-0.91 | 0.27 |  |  |
|  |  |  | 17 | 0.33 | 0.19-0.51 | 0.89 |  | 0.82-0.93 | 0.22 |  |  |
|  |  | GAD-2 | 2 | 1.00 | 0.89-1.00 | 0.24 |  | 0.18-0.33 | 0.24 | 0.76 | 0.68-0.84 |
|  |  |  | 3 | 0.93 | 0.79-0.98 | 0.43 |  | 0.35-0.52 | 0.36 |  |  |
|  |  |  | 4 | 0.83 | 0.66-0.93 | 0.61 |  | 0.53-0.69 | 0.44 |  |  |
|  |  |  | 5 | 0.60 | 0.42-0.75 | 0.74 |  | 0.66-0.81 | 0.34 |  |  |
|  |  |  | 6 | 0.37 | 0.22-0.55 | 0.86 |  | 0.79-0.91 | 0.23 |  |  |
|  |  | GAD-SI | 1 | 1.00 | 0.89-1.00 | 0.16 |  | 0.10-0.23 | 0.16 | 0.78 | 0.69-0.86 |
|  |  |  | 2 | 0.93 | 0.79-0.98 | 0.45 |  | 0.37-0.54 | 0.38 |  |  |
|  |  |  | 3 | 0.70 | 0.52-0.83 | 0.76 |  | 0.67-0.82 | 0.46 |  |  |
| Gibbons et al., 2014 | | CAT-ANX | -0.5 (non-psychiatric sample as comparator) | 0.65 | **-** | 0.93 |  | **-** | 0.58 | **-** | **-** |
|  |  |  | -0.85 (non-psychiatric sample as comparator) | 0.86 | **-** | 0.86 |  | **-** | 0.72 | **-** | **-** |
|  |  |  | -0.5 (entire sample) | 0.67 | **-** | 0.87 |  | **-** | 0.54 | **-** | **-** |
|  |  |  | -0.85 (entire sample) | 0.89 | **-** | 0.77 |  | **-** | 0.66 | **-** | **-** |
| Graham et al., 2019 | | CAT-ANX | - | **-** | **-** | **-** |  | **-** | - | 0.93 | 0.90-0.97 |
| Kertz et al., 2013 | | GAD-7 | 10 | 0.83 | **-** | 0.46 |  | **-** | 0.29 | 0.65 | 0.59-0.73 |
|  |  |  | 11 | 0.77 | **-** | 0.50 |  | **-** | 0.27 |  |  |
|  |  |  | 12 | 0.74 | **-** | 0.55 |  | **-** | 0.29 |  |  |
|  |  |  | 13 | 0.64 | **-** | 0.57 |  | **-** | 0.21 |  |  |
|  |  |  | 14 | 0.59 | **-** | 0.62 |  | **-** | 0.21 |  |  |
|  |  |  | 15 | 0.54 | **-** | 0.69 |  | **-** | 0.23 |  |  |
|  |  |  | 16 | 0.48 | **-** | 0.73 |  | **-** | 0.21 |  |  |
|  |  |  | 17 | 0.41 | **-** | 0.76 |  | **-** | 0.17 |  |  |
| Kim et al., 2021 | | MHS: A | 15 | 0.98 | **-** | 0.80 |  | **-** | 0.78 | 0.95 | **-** |
| Meuldijk et al., 2017 | | WSQ | Q3≥2 | 0.66 | **-** | 0.90 |  | **-** | 0.56 | 0.89 | **-** |
| Munoz-Navarro et al., 2017 | | GAD-7 | 8 | 0.93 | 0.87-0.96 | 0.61 |  | 0.46-0.74 | 0.54 | 0.86 | - |
|  |  |  | 9 | 0.91 | 0.84-0.94 | 0.71 |  | 0.56-0.82 | 0.62 |  |  |
|  |  |  | 10 | 0.87 | 0.80-0.92 | 0.78 |  | 0.63-0.88 | 0.65 |  |  |
|  |  |  | 11 | 0.76 | 0.68-0.82 | 0.78 |  | 0.63-0.88 | 0.54 |  |  |
|  |  |  | 12 | 0.72 | 0.63-0.78 | 0.80 |  | 0.66-0.90 | 0.52 |  |  |
|  |  |  | 13 | 0.68 | 0.60-0.75 | 0.85 |  | 0.72-0.93 | 0.53 |  |  |
|  |  |  | 14 | 0.59 | 0.51-0.67 | 0.88 |  | 0.74-0.95 | 0.47 |  |  |
| Nguyen et al., 2015 | | e-PASS | - | 0.78 | 0.62-0.88 | 0.68 |  | 0.59-0.76 | 0.46 | - | - |
| Rogers et al., 2021 | | CMFC initial screen | - | 0.93 | 0.83-0.98 | 0.63 |  | 0.56-0.72 | 0.56 | - | - |
|  |  | CMFC SAM | - | 0.73 | 0.60-0.84 | 0.89 |  | 0.83-0.93 | 0.62 | - | - |
|  |  | CMFC SAM (preliminary) | - | 0.90 | 0.76-0.96 | 0.86 |  | 0.79-0.91 | 0.76 | - | - |
|  | **Panic disorder** | | | | | | | | | | |
| Ballester et al., 2019 | | WMH-ICS survey (12 months) | 1 | 0.45 | - | 0.98 |  | - | 0.43 | 0.71 | - |
|  | |  | 2 | 0.45 | - | 0.98 |  | - | 0.43 | 0.71 | - |
|  | |  | 3 | 0.45 | - | 0.98 |  | - | 0.43 | 0.71 | - |
|  | |  | 4 | 0.40 | - | 0.98 |  | - | 0.38 | 0.69 | - |
|  | |  | 5 | 0.18 | - | 0.99 |  | - | 0.17 | 0.58 | - |
|  | |  | 6 | 0.18 | - | 1.00 |  | - | 0.18 | 0.59 | - |
|  | |  | 7 | 0.05 | - | 1.00 |  | - | 0.05 | 0.52 | - |
|  | | WMH-ICS survey (lifetime) | 1 | 0.71 | - | 0.83 |  | - | 0.54 | 0.77 | - |
|  | |  | 2 | 0.71 | - | 0.83 |  | - | 0.54 | 0.77 | - |
|  | |  | 3 | 0.71 | - | 0.83 |  | - | 0.54 | 0.77 | - |
|  | |  | 4 | 0.36 | - | 0.88 |  | - | 0.24 | 0.62 | - |
|  | |  | 5 | 0.20 | - | 0.94 |  | - | 0.14 | 0.57 | - |
|  | |  | 6 | 0.07 | - | 0.98 |  | - | 0.05 | 0.52 | - |
|  | |  | 7 | 0.03 | - | 1.00 |  | - | 0.03 | 0.51 | - |
| Donker et al., 2009 | | WSQ | Q4≥1 | 0.90 | - | 0.44 |  | - | 0.34 | 0.76 | 0.59-0.93 |
|  | | PDSS | - | - | - | - |  | - | - | 0.70 | 0.57-0.88 |
| Donker et al., 2011 | | GAD-7 | 5 | 1.00 | 0.68-1.00 | 0.17 |  | 0.12-0.24 | 0.17 | 0.62 | 0.44-0.79 |
|  |  |  | 7 | 0.88 | 0.53-0.98 | 0.32 |  | 0.25-0.39 | 0.20 |  |  |
|  |  |  | 9 | 0.88 | 0.53-0.98 | 0.37 |  | 0.30-0.45 | 0.25 |  |  |
|  |  |  | 10 | 0.75 | 0.41-0.93 | 0.44 |  | 0.37-0.52 | 0.19 |  |  |
|  |  |  | 11 | 0.63 | 0.31-0.86 | 0.50 |  | 0.42-0.58 | 0.13 |  |  |
|  |  |  | 12 | 0.50 | 0.22-0.78 | 0.56 |  | 0.48-0.63 | 0.06 |  |  |
|  |  |  | 13 | 0.50 | 0.22-0.78 | 0.62 |  | 0.54-0.70 | 0.12 |  |  |
|  |  |  | 14 | 0.50 | 0.22-0.78 | 0.69 |  | 0.61-0.76 | 0.19 |  |  |
|  |  |  | 15 | 0.38 | 0.14-0.69 | 0.75 |  | 0.68-0.81 | 0.13 |  |  |
|  |  |  | 16 | 0.25 | 0.07-0.59 | 0.83 |  | 0.75-0.87 | 0.08 |  |  |
|  |  |  | 17 | 0.25 | 0.07-0.95 | 0.85 |  | 0.79-0.90 | 0.10 |  |  |
|  |  | GAD-2 | 2 | 1.00 | 0.68-1.00 | 0.21 |  | 0.15-0.28 | 0.21 | 0.64 | 0.46-0.83 |
|  |  |  | 3 | 0.75 | 0.41-0.93 | 0.37 |  | 0.30-0.45 | 0.12 |  |  |
|  |  |  | 4 | 0.63 | 0.31-0.86 | 0.54 |  | 0.46-0.62 | 0.17 |  |  |
|  |  |  | 5 | 0.50 | 0.22-0.78 | 0.68 |  | 0.61-0.75 | 0.18 |  |  |
|  |  |  | 6 | 0.38 | 0.14-0.69 | 0.83 |  | 0.76-0.89 | 0.21 |  |  |
|  |  | GAD-SI | 2 | 0.88 | 0.52-0.98 | 0.39 |  | 0.31-0.47 | 0.27 | 0.65 | 0.49-0.82 |
|  |  |  | 3 | 0.50 | 0.22-0.78 | 0.68 |  | 0.50-0.75 | 0.18 |  |  |
| Meuldijk et al., 2017 | | WSQ | Q4≥1 | 0.81 | - | 0.95 |  | - | 0.76 | 0.98 | - |
| Nguyen et al., 2015 | | e-PASS | - | 0.71 | 0.55-0.84 | 0.91 |  | 0.85-0.95 | 0.62 | - | - |
| Oromendia et al., 2015 | | WSQ | 0 | 1.00 | - | 0.00 |  | - | 0.00 | 0.82 | 0.74-0.90 |
|  |  |  | 1 | 0.83 | - | 0.74 |  | - | 0.57 |  |  |
|  |  |  | 2 | 0.81 | - | 0.80 |  | - | 0.61 |  |  |
|  |  |  | 3 | 0.39 | - | 0.92 |  | - | 0.31 |  |  |
|  |  |  | 4 | 0.19 | - | 0.98 |  | - | 0.17 |  |  |
|  |  |  | 5 | 0.00 | - | 1.00 |  | - | 0.00 |  |  |
| Rogers et al., 2021 | | CMFC initial screen | - | 0.79 | 0.59-0.91 | 0.52 |  | 0.45-0.59 | 0.31 | - | - |
|  |  | CMFC SAM | - | 0.32 | 0.28-0.66 | 0.76 |  | 0.69-0.81 | 0.08 | - | - |
|  | **Social phobia** | | | | | | | | | | |
| Donker et al., 2009 | | WSQ | Q8=1 & Q9=1 | 0.72 | - | 0.73 |  | - | 0.45 | 0.72 | 0.62-0.82 |
|  | | FQ | - | - | - | - |  | - | - | 0.82 | 0.74-0.89 |
| Donker et al., 2011 | | GAD-7 | 5 | 0.85 | 0.66-0.94 | 0.16 |  | 0.11-0.23 | 0.01 | 0.57 | 0.45-0.7 |
|  |  |  | 7 | 0.73 | 0.54-0.86 | 0.31 |  | 0.24-0.40 | 0.04 |  |  |
|  |  |  | 9 | 0.69 | 0.50-0.84 | 0.37 |  | 0.29-0.45 | 0.06 |  |  |
|  |  |  | 10 | 0.62 | 0.43-0.78 | 0.44 |  | 0.36-0.53 | 0.06 |  |  |
|  |  |  | 11 | 0.58 | 0.39-0.74 | 0.50 |  | 0.42-0.50 | 0.08 |  |  |
|  |  |  | 12 | 0.54 | 0.35-0.71 | 0.57 |  | 0.49-0.65 | 0.11 |  |  |
|  |  |  | 13 | 0.42 | 0.26-0.61 | 0.63 |  | 0.54-0.70 | 0.05 |  |  |
|  |  |  | 14 | 0.42 | 0.26-0.61 | 0.70 |  | 0.62-0.77 | 0.12 |  |  |
|  |  |  | 15 | 0.38 | 0.22-0.57 | 0.77 |  | 0.69-0.83 | 0.15 |  |  |
|  |  |  | 16 | 0.27 | 0.14-0.46 | 0.83 |  | 0.76-0.89 | 0.10 |  |  |
|  |  |  | 17 | 0.19 | 0.09-0.38 | 0.86 |  | 0.78-0.91 | 0.05 |  |  |
|  |  | GAD-2 | 2 | 0.85 | 0.66-0.94 | 0.21 |  | 0.15-0.28 | 0.06 | 0.57 | 0.45-0.68 |
|  |  |  | 3 | 0.73 | 0.54-0.86 | 0.38 |  | 0.30-0.47 | 0.11 |  |  |
|  |  |  | 4 | 0.58 | 0.39-0.74 | 0.55 |  | 0.46-0.63 | 0.13 |  |  |
|  |  |  | 5 | 0.46 | 0.29-0.65 | 0.70 |  | 0.62-0.77 | 0.16 |  |  |
|  |  |  | 6 | 0.15 | 0.06-0.34 | 0.81 |  | 0.73-0.87 | 0.04 |  |  |
|  |  | GAD-SI | 2 | 0.69 | 0.50-0.84 | 0.39 |  | 0.31-0.47 | 0.08 | 0.76 | 0.58-0.94 |
|  |  |  | 3 | 0.35 | 0.19-0.54 | 0.67 |  | 0.59-0.75 | 0.02 |  |  |
| Meuldijk et al., 2017 | | WSQ | Q8=1 & Q9=1 | 0.79 | - | 0.93 |  | - | 0.72 | 0.95 | - |
| Nguyen et al., 2015 | | e-PASS | - | 0.60 | 0.47-0.71 | 0.90 |  | 0.84-0.96 | 0.50 | - | - |
| Rogers et al., 2021 | | CMFC initial screen | - | 0.92 | 0.72-0.99 | 0.53 |  | 0.46-0.60 | 0.45 | - | - |
|  |  | CMFC SAM | - | 0.42 | 0.23-0.63 | 0.75 |  | 0.68-0.80 | 0.17 | - | - |
|  | **Post-traumatic stress disorder** | | | | | | | | | | |
| Donker et al., 2009 | | WSQ | Q10=1 or Q11=1 | 0.83 | - | 0.47 |  | - | 0.30 | 0.65 | 0.51-0.80 |
|  |  | IES | - | - | - | - |  | - | - | 0.82 | 0.67-0.97 |
| Donker et al., 2011 | | GAD-7 | 5 | 1.00 | 0.68-1.00 | 0.17 |  | 0.12-0.24 | 0.17 | 0.76 | 0.58-0.94 |
|  |  |  | 7 | 0.88 | 0.53-0.98 | 0.32 |  | 0.25-0.39 | 0.20 |  |  |
|  |  |  | 9 | 0.88 | 0.53-0.98 | 0.37 |  | 0.29-0.45 | 0.25 |  |  |
|  |  |  | 10 | 0.88 | 0.53-0.98 | 0.45 |  | 0.37-0.53 | 0.33 |  |  |
|  |  |  | 11 | 0.88 | 0.53-0.98 | 0.51 |  | 0.43-0.59 | 0.39 |  |  |
|  |  |  | 12 | 0.75 | 0.41-0.93 | 0.57 |  | 0.49-0.65 | 0.32 |  |  |
|  |  |  | 13 | 0.75 | 0.41-0.94 | 0.64 |  | 0.56-0.71 | 0.39 |  |  |
|  |  |  | 14 | 0.75 | 0.41-0.95 | 0.71 |  | 0.63-0.77 | 0.46 |  |  |
|  |  |  | 15 | 0.75 | 0.41-0.96 | 0.77 |  | 0.70-0.83 | 0.52 |  |  |
|  |  |  | 16 | 0.63 | 0.31-0.68 | 0.84 |  | 0.77-0.89 | 0.47 |  |  |
|  |  |  | 17 | 0.38 | 0.14-0.69 | 0.86 |  | 0.79-0.91 | 0.24 |  |  |
|  |  | GAD-2 | 2 | 0.88 | 0.52-0.98 | 0.20 |  | 0.14-0.27 | 0.08 | 0.74 | 0.57-0.92 |
|  |  |  | 3 | 0.88 | 0.52-0.98 | 0.38 |  | 0.30-0.46 | 0.26 |  |  |
|  |  |  | 4 | 0.88 | 0.52-0.98 | 0.55 |  | 0.47-0.63 | 0.43 |  |  |
|  |  |  | 5 | 0.88 | 0.52-0.98 | 0.71 |  | 0.63-0.77 | 0.59 |  |  |
|  |  |  | 6 | 0.38 | 0.14-0.69 | 0.83 |  | 0.76-0.88 | 0.21 |  |  |
|  |  | GAD-SI | 2 | 0.88 | 0.53-0.98 | 0.39 |  | 0.31-0.47 | 0.27 | 0.69 | 0.53-0.86 |
|  |  |  | 3 | 0.63 | 0.31-0.86 | 0.69 |  | 0.61-0.75 | 0.32 |  |  |
| Gaynes et al., 2010 | | M-3 | 2 | 0.88 | 0.74-0.96 | 0.70 |  | 0.73-0.80 | 0.58 | - | - |
| Meuldijk et al., 2017 | | WSQ | Q10=1 or Q11=1 | 0.79 | - | 0.52 |  | - | 0.31 | 0.86 | - |
| Nguyen et al., 2015 | | e-PASS | - | 0.75 | 0.47-0.91 | 0.92 |  | 0.87-0.96 | 0.67 | - | - |
|  | **Obsessive compulsive disorder** | | | | | | | | | | |
| Donker et al., 2009 | | WSQ | Q12≥1 | 0.80 | - | 0.69 |  | - | 0.49 | 0.81 | 0.65-0.97 |
|  |  | YBOCS | - | - | - | - |  | - | - | 0.86 | 0.72-0.99 |
| Meuldijk et al., 2017 | | WSQ | Q12≥1 | 0.67 | - | 0.91 |  | - | 0.58 | 0.82 | - |
| Nguyen et al., 2015 | | e-PASS | - | 0.75 | 0.47-0.91 | 0.92 |  | 0.87-0.96 | 0.67 | - | - |
|  | **Agoraphobia** | | | | | | | | | | |
| Donker et al., 2009 | | WSQ | Q5=1 | 1.00 | - | 0.63 |  | - | 0.63 | 0.81 | 0.73-0.90 |
|  |  | FQ | - | - | - | - |  | - | - | 0.81 | 0.70-0.91 |
| Meuldijk et al., 2017 | | WSQ | Q5=1 | 0.81 | - | 0.95 |  | - | 0.76 | 0.80 | - |
|  | **Major depressive disorder** | | | | | | | | | | |
| Achtyes et al., 2015 | | CAD-MDD (entire sample) | - | 0.96 | - | 0.64 |  | **-** | 0.60 | **-** | **-** |
|  |  | CAD-MDD (non-psychiatric sample as comparator) | - | 0.96 | - | 1.00 |  | **-** | 0.96 | **-** | **-** |
| Cano-Vindel et al., 2018 | | PHQ-2 | 0 | 1.00 | 0.97-1.00 | 0.00 |  | 0.00-0.07 | 0.00 | - | - |
|  |  |  | 1 | 1.00 | 0.97-1.00 | 0.13 |  | 0.07-0.27 | 0.13 | - | - |
|  |  |  | 2 | 1.00 | 0.97-1.00 | 0.27 |  | 0.16-0.40 | 0.27 | - | - |
|  |  |  | 3 | 0.90 | 0.84-0.94 | 0.61 |  | 0.47-0.73 | 0.51 | - | - |
|  |  |  | 4 | 0.78 | 0.70-0.84 | 0.73 |  | 0.60-0.84 | 0.51 | - | - |
|  |  |  | 5 | 0.56 | 0.47-0.64 | 0.94 |  | 0.83-0.98 | 0.50 | - | - |
|  |  |  | 6 | 0.39 | 0.31-0.48 | 0.96 |  | 0.86-0.99 | 0.35 | - | - |
| Du et al., 2017 | | PHQ-9 | 5 | 0.95 | **-** | 0.53 |  | **-** | 0.48 | 0.90 | 0.82-0.97 |
|  |  |  | 6 | 0.95 | **-** | 0.65 |  | **-** | 0.60 |  |  |
|  |  |  | 7 | 0.95 | **-** | 0.69 |  | **-** | 0.64 |  |  |
|  |  |  | 8 | 0.89 | **-** | 0.74 |  | **-** | 0.63 |  |  |
|  |  |  | 9 | 0.89 | **-** | 0.79 |  | **-** | 0.68 |  |  |
|  |  |  | 10 | 0.74 | **-** | 0.85 |  | **-** | 0.59 |  |  |
|  |  |  | 11 | 0.63 | **-** | 0.92 |  | **-** | 0.55 |  |  |
|  |  |  | 12 | 0.53 | **-** | 0.94 |  | **-** | 0.47 |  |  |
|  |  |  | 13 | 0.47 | **-** | 0.94 |  | **-** | 0.41 |  |  |
|  |  |  | 14 | 0.42 | **-** | 0.97 |  | **-** | 0.39 |  |  |
|  |  |  | 15 | 0.37 | **-** | 0.97 |  | **-** | 0.34 |  |  |
| Gibbons et al., 2012 | | CAT-DI | 0.28 | 0.98 | **-** | 0.63 |  | **-** | 0.61 | **-** | **-** |
|  |  |  | -0.19 | 0.82 | **-** | 0.85 |  | **-** | 0.67 | **-** | **-** |
| Gibbons et al., 2013 | | CAD-MDD | - | 0.95 | **-** | 0.87 |  | **-** | 0.82 | **-** | **-** |
|  | |  | (cross-validated) | 0.94 | **-** | 0.82 |  | **-** | 0.76 | **-** | **-** |
| Graham et al., 2019 | | CAD-MDD | 50 | 0.77 | **-** | 0.93 |  | **-** | 0.70 | 0.85 | 0.76-0.94 |
| Nguyen et al., 2015 | | e-PASS | - | 0.86 | 0.73-0.94 | 0.79 |  | 0.71-0.85 | 0.65 | - | - |
| Nielsen et al., 2017 | | MID | 20 | 0.62 | 0.53-0.71 | 0.63 |  | 0.35-0.85 | 0.25 | 0.66 | 0.52-0.81 |
|  |  |  | 20–24 | 0.20 | 0.05-0.72 | 0.83 |  | 0.52-0.98 | 0.03 | - | - |
|  |  |  | 25–29 | 0.21 | 0.60-0.46 | 0.81 |  | 0.58-0.95 | 0.02 | - | - |
|  |  |  | 30 | 0.41 | 0.31-0.52 | 0.85 |  | 0.72-0.94 | 0.26 | 0.72 | 0.63-0.81 |
|  | **Bipolar disorder/Bipolar spectrum disorder** | | | | | | | | | | |
| Gaynes et al., 2010 | | M-3 | 2 | 0.88 | 0.77-0.95 | 0.70 |  | 0.66-0.74 | 0.58 | - | - |
| Rogers et al., 2021 | | CMFC initial screen | - | 0.63 | 0.36-0.84 | 0.79 |  | 0.60-0.90 | 0.42 | - | - |
|  |  | CMFC SAM | - | 0.50 | 0.26-0.75 | 0.97 |  | 0.82-1.00 | 0.47 | - | - |
|  | | **Attention deficit hyperactivity disorder** | | | | | | | | | |
| Rogers et al., 2021 | | CMFC initial screen | - | 0.94 | 0.80-0.99 | 0.61 |  | 0.53-0.67 | 0.55 | - | - |
|  |  | CMFC SAM | - | 0.69 | 0.52-0.83 | 0.86 |  | 0.79-0.90 | 0.55 | - | - |
|  | **Alcohol use disorder** | | | | | | | | | | |
| Donker et al., 2009 | | WSQ | Q13≥2 & Q14≥3 | 0.83 | **-** | 0.72 |  | **-** | 0.55 | 0.77 | 0.68-0.86 |
|  |  | AUDIT | - | - | **-** | - |  | **-** | - | 0.75 | 0.66-0.84 |
| McNeely et al., 2015 | | SISQ | 1 | 0.87 | 0.75-0.94 | 0.74 |  | 0.70-0.78 | 0.61 | 0.80 | 0.76-0.85 |
| Meuldijk et al., 2017 | | WSQ | Q13≥2 & Q14≥3 | 0.56 | **-** | 0.92 |  | **-** | 0.48 | 0.82 | **-** |
| Nguyen et al., 2015 | | e-PASS | - | 0.43 | 0.16-0.75 | 1.00 |  | 0.98-1.00 | 0.43 | - | - |
| Sanchez et al, 2021 | | TAPS-1 | Daily/almost daily | 0.97 | - | 0.99 |  | **-** | 0.96 | **-** | **-** |
|  | **Substance use disorder** | | | | | | | | | | |
| McNeely et al., 2015 | | SISQ | 1 | 0.85 | 0.75-0.92 | 0.89 |  | 0.85-0.92 | 0.74 | 0.87 | 0.83-0.91 |
| Rogers et al., 2021 | | CMFC initial screen | - | 0.80 | 0.60-0.90 | 0.92 |  | 0.87-0.95 | 0.72 | - | - |
|  |  | CMFC SAM | - | 0.67 | 0.48-0.81 | 0.96 |  | 0.91-0.98 | 0.63 | - | - |
|  | **Anorexia nervosa** | | | | | | | | | | |
| ter Huurne et al., 2015 | | EDQ-O | - | 0.44 | **-** | 1.00 |  | **-** | 0.44 | 0.72 | **-** |
|  | **Bulimia nervosa** | | | | | | | | | | |
| Nguyen et al., 2015 | | e-PASS | - | 0.50 | 0.24-0.76 | 0.97 |  | 0.92-0.99 | 0.47 | - | - |
| ter Huurne et al., 2015 | | EDQ-O | - | 0.78 | **-** | 0.88 |  | **-** | 0.66 | 0.83 | **-** |
|  | **Binge eating disorder** | | | | | | | | | | |
| ter Huurne et al., 2015 | | EDQ-O | - | 0.66 | **-** | 0.98 |  | **-** | 0.64 | 0.82 | **-** |
|  | **Eating disorder not otherwise specified** | | | | | | | | | | |
| ter Huurne et al., 2015 | | EDQ-O | - | 0.87 | **-** | 0.72 |  | **-** | 0.59 | 0.79 | **-** |
|  | **Emotionally unstable personality disorder** | | | | | | | | | | |
| Folwer et al., 2018 | | FFM composites: Neuroticism + agreeableness | 112 | 0.70 | **-** | 0.62 |  | **-** | 0.32 | 0.72 | **-** |
|  |  | FFM composites: Neuroticism + agreeableness + Conscientiousness | 185 | 0.71 | **-** | 0.62 |  | **-** | 0.33 | 0.73 | **-** |
|  | | SCIDII-PQ | 8 | 0.78 | **-** | 0.80 |  | **-** | 0.58 | 0.86 | **-** |
|  | | PID-5 | 11 | 0.81 | **-** | 0.76 |  | **-** | 0.57 | 0.87 | **-** |
| Lohanan et al., 2020 | | SI-Board | 0 | 1.00 | 0.79-1.00 | 0.00 |  | 0.00-0.07 | 0.00 | 0.83 | 0.72-0.91 |
|  |  |  | 1 | 1.00 | 0.79-1.00 | 0.08 |  | 0.02-0.19 | 0.08 |  |  |
|  |  |  | 2 | 1.00 | 0.79-1.00 | 0.15 |  | 0.07-0.28 | 0.15 |  |  |
|  |  |  | 3 | 1.00 | 0.79-1.00 | 0.21 |  | 0.11-0.35 | 0.21 |  |  |
|  |  |  | 4 | 0.94 | 0.70-1.00 | 0.40 |  | 0.27-0.55 | 0.34 |  |  |
|  |  |  | 5 | 0.94 | 0.70-1.00 | 0.50 |  | 0.36-0.64 | 0.44 |  |  |
|  |  |  | 6 | 0.75 | 0.48-0.93 | 0.63 |  | 0.49-0.76 | 0.38 |  |  |
|  |  |  | 7 | 0.75 | 0.48-0.93 | 0.73 |  | 0.59-0.84 | 0.48 |  |  |
|  |  |  | 8 | 0.63 | 0.35-0.85 | 0.83 |  | 0.70-0.92 | 0.46 |  |  |
|  |  |  | 9 | 0.56 | 0.30-0.80 | 0.92 |  | 0.82-0.98 | 0.48 |  |  |
|  |  |  | 10 | 0.38 | 0.15-0.65 | 0.96 |  | 0.87-1.00 | 0.34 |  |  |
|  |  |  | 11 | 0.31 | 0.11-0.59 | 0.98 |  | 0.90-1.00 | 0.29 |  |  |
|  |  |  | 12 | 0.13 | 0.02-0.38 | 1.00 |  | 0.93-1.00 | 0.13 |  |  |
|  |  |  | 13 | 0.06 | 0.02-0.30 | 1.00 |  | 0.93-1.00 | 0.06 |  |  |
|  |  |  | 14 | 0.00 | 0.00-0.21 | 1.00 |  | 0.93-1.00 | 0.00 |  |  |
|  | **Psychosis** | | | | | | | | | | |
| Guinart et al., 2021 | | CAT-psychosis (entire sample) | - | - | **-** | - |  | **-** | - | 0.85 | 0.81-0.89 |
|  |  | CAT-psychosis (SCID-I only) | - | - | **-** | - |  | **-** | - | 0.80 | 0.73-0.86 |
|  | | **Suicidality** | | | | | | | | | |
| Rogers et al., 2021 | | CMFC initial screen (thoughts of own death) | - | 0.75 | 0.54-0.96 | 0.89 |  | 0.82-0.96 | 0.64 | - | - |
|  |  | CMFC initial screen (ideation) | - | 0.75 | 0.61-0.90 | 0.84 |  | 0.76-0.92 | 0.59 | - | - |
|  |  | CMFC initial screen (specific plan) | - | 1.00 | 0.73-1.00 | 0.80 |  | 0.71-0.89 | 0.80 | - | - |
| Yoon et al., 2020 | | UBCS | 1 | 0.91 | **-** | 0.85 |  | **-** | 0.76 | - | - |
|  |  |  | 2 | 0.68 | **-** | 0.93 |  | **-** | 0.61 | - | - |
|  |  |  | 3 | 0.50 | **-** | 0.96 |  | **-** | 0.46 | - | - |

***Key.*** AUDIT, Alcohol Use Disorders Identification Test; BSA, Brief Scale for Anxiety; CAD-MDD, Computerized Adaptive Diagnosis for MDD; CAT-ANX, Computerized Adaptive Test – Anxiety; CAT-DI, Computerized Adaptive Test – Depression Inventory; CAT-MANIA, Computerized Adaptive Test – Mania; CAT-Psychosis, Computerized Adaptive Test – Psychosis; CES-D, Center for Epidemiological Studies – Depression Scale; CMFC, Connected Mind Fast Check; EDQ-O, Eating Disorder Questionnaire-Online; e-PASS, electronic psychological assessment screening system; FFM, Five Factor Model questionnaire; FQ, Fear Questionnaire; GAD-2, 2-item Generalized Anxiety Disorder scale; GAD-7, 7-item Generalized Anxiety Disorder scale; GAD-SI, single-item Generalized Anxiety Disorder scale; IES-R, Impact of Event Scale-Revised; K10, Kessler Psychological Distress Scale; M-3, My Mood Monitor; MDI, Major Depression Inventory; MHS: A, Mental Health Screening Tool for Anxiety Disorders; PAI, Panic Appraisal Inventory; PDSS, Panic Disorder Severity Scale; PHQ-2, 2-item Patient Health Questionnaire; PHQ-9, 9-item Patient Health Questionnaire; PID-5, Personality Inventory for DSM-5; PI-R, PADUA Inventory Revised; PSWQ, Penn State Worry Questionnaire; SAM, Standardized Assessment Module; SCID-I, Structured Clinical Interview for DSM Axis I disorders; SCID-II-PQ, SCID-II Personality Questionnaire; SIAS, Social Interaction and Anxiety Scale; SI-Board, Screening Instrument for borderline personality disorder; SID, single item depression scale; SISQ, single item screening question; SPS, Social Phobia Scale; TAPS-1, Tobacco, Alcohol, Prescription Medication, and Other Substance use scale; UBCS, Ultra Brief Checklist for Suicidality; WDQ, Worry Domains Questionnaire; WMH-ICS, WHO World Mental Health International College Student; WSQ, Web-based Screening Questionnaire; YBOCS, Yale-Brown Obsessive Compulsive Scale
